# Transferrin Receptor Functionally Marks Thermogenic Adipocytes

**DOI:** 10.3389/fcell.2020.572459

**Published:** 2020-11-05

**Authors:** Jin Qiu, Zhiyin Zhang, Sainan Wang, Yanru Chen, Caizhi Liu, Sainan Xu, Dongmei Wang, Junlei Su, Mengshan Ni, Jian Yu, Xiangdi Cui, Lu Ma, Tianhui Hu, Yepeng Hu, Xuejiang Gu, Xinran Ma, Jiqiu Wang, Lingyan Xu

**Affiliations:** ^1^Shanghai Key Laboratory of Regulatory Biology, Institute of Biomedical Sciences, School of Life Sciences, East China Normal University, Shanghai, China; ^2^Department of Endocrinology and Metabolism, China National Research Center for Metabolic Diseases, Ruijin Hospital, Shanghai Jiao Tong University School of Medicine, Shanghai, China; ^3^Department of Endocrine and Metabolic Diseases, the First Affiliated Hospital of Wenzhou Medical University, Wenzhou, China

**Keywords:** Tfr1, iron homeostasis, thermogenic adipocytes, brown gene program, mitochondrial integrity

## Abstract

**Background:**

Thermogenic adipocytes, including beige and brown adipocytes, are critical for thermogenesis and energy homeostasis. Identification of functional cell surface markers of thermogenic adipocytes is of significance for potential application in biological and clinical practices.

**Methods:**

With a combination of RNA-sequencing of *in vivo* and *in vitro* models, we identified transferrin receptor (Tfr1), a receptor specialized for cellular iron uptake, as a previously unappreciated cell surface molecule for thermogenic adipocytes compared to white adipocytes. The alternation of Tfr1 levels under physiological and pathological stimuli was assessed, and the mitochondria functionality, browning capacity, and iron metabolism of mature adipocytes were examined with *Tfr1* knockdown.

**Results:**

Tfr1 was expressed predominantly in thermogenic adipocytes versus white adipocyte, and its expression levels were tightly correlated with the activation or inhibition status of thermogenic adipocytes under external stimuli. Besides, *Tfr1* gene deficiency in thermogenic adipocytes led to reduced thermogenic gene programs and mitochondrial integrity.

**Conclusion:**

Tfr1 functionally marks thermogenic adipocytes and could serve as a potential thermogenic adipocyte surface marker.

## Introduction

The obesity pandemic in modern society predisposes a large population to metabolic diseases, including Type 2 diabetes, hepatic steatosis, and cardiovascular diseases, thus greatly burdening the healthcare system (Jung and Choi, 2014; Chung et al., 2016). Obesity is manifested as excess accumulation of fat in adipose tissues. Three classes of adipose tissues were identified, featuring distinct location, morphology, and function (Hildebrand et al., 2018). White adipose tissue is a major site for energy storage in the form of triglyceride, while brown adipose tissue (BAT) is thermogenically poised to consume fuel for thermogenesis and energy expenditure. Beige adipocytes, a newly discovered type of adipose tissue, reside heterogeneously with white adipocytes in certain white fat depots. It resembles white adipocyte in resting state, while becoming highly inducible upon cold or β-adrenergic signaling activation and retains thermogenic capacity similar to brown adipocyte through a process referred to as “browning of white fat.” The functions of these three kinds of adipose tissues balance energy depository and expenditure, thus determining overall body weight and energy homeostasis (Scheja and Heeren, 2016; Brandão et al., 2017).

Recently, research interests have been piqued toward the thermogenic adipocytes including brown and beige adipocytes for their potent capability in dissipating excessive calories upon activation. In addition, brown and beige adipocytes consume large amounts of lipid and glucose as fuels for thermogenesis, thus serving as a metabolic sink critical for maintaining blood lipid/glucose levels (Sidossis and Kajimura, 2015). Evidences from genetic animal models attest to the crucial protective roles of thermogenic adipocytes against obesity and metabolic homeostasis. For example, genetic ablation of critical thermogenic genes *Prdm16* and *Pgc1*α led to obesity and metabolic dysfunctions in mice (Gastaldi et al., 2007; Hasegawa et al., 2018). Importantly, in adult humans, functional brown and beige fat exist as a heterogeneous mixture located in deeper cervical, supraclavicular, and paraspinal areas, the exact constituents of which are dependent on their depth under the skin (Scheele et al., 2014). Moreover, white depots in humans also possess browning ability upon stimulation. For example, pheochromocytoma patients usually feature a pathologically lean status because of the strong white fat browning in various white depots caused by the sustained elevation of β3-adrenergic signaling (Wang et al., 2011; Frontini et al., 2013). Importantly, the ability of brown and beige fat activation significantly declined in obese or elderly subjects (Diaz et al., 2014), indicative of the potential of targeting thermogenic adipocytes to prevent and treat obesity or metabolic dysfunctions in humans. To date, cold and β3-androgenic agonists, i.e., Mirabegron, have been shown to activate thermogenic adipocytes in humans, but their wide applications have been hindered by side effects on cardiovascular systems (Ng et al., 2017; Baskin et al., 2018), warranting continued search for targeting strategies on thermogenic adipocytes.

It would be critical to take a few caveats into consideration when designing novel strategies to activate thermogenic adipocytes. For example, since white and brown/beige adipocytes share many common protein expressions, molecules specific to brown/beige adipocytes need to be identified to ensure target specificity and avoid undesired effects on white adipocytes. Hitherto, a number of genes have been identified that featured distinct expressions in thermogenic adipocytes compared to white adipocyte, including *Tbx1* and *Tmem26* for beige fat; uncoupling protein 1 (*Ucp1*), *Cidea*, and *Prdm16* for brown fat; and *Pat2* and *P2rx5* for brown/beige fat (Ussar et al., 2014; De Jong et al., 2015; Rockstroh et al., 2015). However, these markers either have intracellular expressions that limited their implications in intact tissues or *in vivo* studies, or are surface markers without functionality evaluations. In this sense, it would be vital to identify novel cell surface molecules of thermogenic adipocytes that could impact thermogenic functions.

In the present study, we sought to identify cell surface molecules for thermogenic adipocytes versus white adipocytes using a combination of RNA-sequencing analysis, *in vitro* assays, and *in vivo* approaches. We unveiled that the surface molecule transferrin receptor (Tfr1) features high expressions in thermogenic adipocytes versus white adipocytes in cells and rodents. Tfr1, as a membrane protein, plays critical roles in iron homeostasis and is involved in various physiological and pathological processes by formation of the Tfr1–Transferrin (Tfr)–iron complex, internalization into endosomes, and disassociation from iron in acidification to eventually release iron into the cytosol via Divalent metal transporter 1 (Dmt1) on the endosomal membrane (Touret et al., 2003). In this work, we found that Tfr1 expression level is tightly correlated with metabolic status, and it is indispensable for intact thermogenic and mitochondrial programs in brown/beige adipocytes. Thus, we proposed Tfr1 as a promising therapeutic cell surface target for obesity and metabolic diseases.

## Materials and Methods

### Preadipocyte Isolation and Differentiation

Adipocytes and stromal vascular fraction (SVF) from mice epididymal, subcutaneous, and brown fat were separated as described previously (Lavery et al., 2016). Briefly, tissue was fractionated by collagenase for 30 min at 37°C immediately after excision. The released cells were sieved through a 200-μm polypropylene filter. Floating cells (adipocytes) were transferred to a 2-ml tube, washed in 1 ml of Hanks’ Balanced Salt Solution (HBSS, 14025092, Gibco, Germany) with 5% bovine serum albumin (BSA, SRE0096, Sigma-Aldrich, United States), and collected after floating. The non-floating cells (SVF) were centrifuged at 3000 rpm for 5 min, and the supernatant was removed. The SVFs were cultured and differentiated following a standard protocol. Briefly, after reaching confluence (day 0), differentiation was initiated by adding differentiation medium containing 5 μg/ml insulin (HI0240, Eli Lilly, United States), 0.5 mM isobutylmethylxanthine (I7018, Sigma, United States), 1 μM dexamethasone (D4902, Sigma, United States), 1 nM T3 (T2877, Sigma, United States), and 1 μM rosiglitazone (R2408, Sigma, United States). After 2 days, the medium was replaced with insulin and T3. Medium was changed every 2 days until day 8. The adipocytes and SVF were kept for further usage. The immortalized beige and brown preadipocytes were kind gifts from the Chinese Academy of Sciences (Prof. Qiurong Ding) and Fudan University Shanghai Medical College (Prof. Dongning Pan).

### RNA-seq and Bioinformatic Analysis

Total RNA was extracted with Trizol (9109, Takara, Japan) and purified by RNeasy Micro kit (74004, QIAGEN, Germany). The quality of purified RNA was examined by NanoDrop ND-1000 and Agilent Bioanalyzer 2100 (Agilent Technologies, Santa Clara, CA, United States). RNA libraries were prepared using the TruSeq RNA Library Preparation Kit (Illumina, San Diego, CA, United States) and were performed on an Illumina HiSeq 2500 sequencing instrument (Illumina, San Diego, CA, United States). The high-quality reads were aligned to the mouse genome (GRCm38/mm10) assembled against mouse mRNA annotation using HTSeq. Differential gene expression was determined using cufflink (version:2.1.1). The fold change was calculated using the ratio of inguinal adipose tissue (iWAT)/epididymal adipose tissue (eWAT) using DESeq2 package. The differentially expressed genes with a *P* less than 0.05 and fold change greater than 2 were considered for further evaluation.

The heatmap was generated by R software (version 3.6.3) and the Venn diagram was performed with the web tool^1^. Selected gene sets were annotated by Gene ontology (GO) analysis (Ashburner et al., 2000), divided into molecular function (MF), biological process (BP), and cellular component (CC), as well as Kyoto Encyclopedia of Genes and Genomes (KEGG) database (Altermann and Klaenhammer, 2005). GO and KEGG enrichment analysis were conducted by DAVID database (DAVID 6.8)^2^ (Huang et al., 2009a,b).

### Mice Experiments

*C57BL/6J* and *129/Sv* mice were maintained on a 12-h light/dark cycle and had free access to food and water. For establishment of animal models, *C57BL/6J* male mice were used in the study. To induce browning of adipose tissues, 8-week-old male mice were housed at either 22°C normal or 5°C cold environment in a thermo-controlled incubator for 1 week. Besides, 4-month-old mice were injected intraperitoneally with either saline or CL316243 (1 μg/g body weight) daily for 14 days, as described previously (Labbe et al., 2016) or intraperitoneally with either saline or Rosiglitazone (10 μg/g body weight) daily for 10 days. To study the effects of obesity and aging on gene expression, 2-month-old male mice with *C57BL/6J* background were fed either chow diet or high-fat diet (HFD) containing 60% fat (Research diet) for 8 weeks. Meanwhile, 5-month-old *db/db*, 18-month-old mice, and their individual controls were also used. All animal experiments were approved by the Animal Care and the Animal Ethics Committee of East China Normal University.

### Real-Time PCR

RNAs from adipocytes and mouse tissues were extracted using Trizol (9109, Takara, Japan). Total RNA (1 μg) was reversely transcribed with PrimeScript RT reagent Kit with gDNA Eraser kit (PR047Q, Takara, Japan) and qPCR was performed using SYBR green (11143ES50, Yeasen, China) on the LightCycler480 system (Roche, Switzerland). mRNA levels were calculated by the ΔΔCT method with the level of 36B4 as the internal control. The primers used for qPCR are in Supplementary Table S1.

### Western Blot and Membrane Fractionation Isolation

For Western blot, the protein concentrations were quantified using a BCA Protein Assay kit (P0010, Beyotime Biotechnology, China), and equal amounts of protein were subjected to 8% sodium dodecyl sulfate polyacrylamide gel electrophoresis (SDS-PAGE) and then transferred to a NC membrane (66485, PALL, United States). After blocking with 5% skimmed milk, the membrane was incubated overnight at 4°C with indicated primary antibodies and subsequently incubated with secondary antibody at room temperature for 1 h. The images of the blots were detected using the Odyssey imaging system (LI-COR Biotechnology, United States) (Yue et al., 2019). Proteins from the membrane and cytoplasm fractions of adipocytes were separated using the Membrane and Cytosol Protein Extraction Kit (P0033, Beyotime Biotechnology, China), according to the manufacturer’s instruction. Briefly, 100 mg of iWAT or BAT were homogenized on ice in Membrane Protein Extraction Reagent A containing proteasome inhibitor cocktail (Sigma). The homogenate was placed on ice for 15 min and centrifuged at 700 × *g* for 10 min, followed by further centrifuge at 14,000 × *g* for 30 min, and supernatant (cytosol protein) was carefully collected and the pellet was then resuspended in Membrane Protein Extraction Reagent B and centrifuged at 14,000 × *g* for 5 min. The supernatant containing cell membrane protein was eventually collected. Antibodies used for Western blot are as follows: anti-Tfr1 (ab84036, Abcam, United Kingdom), anti-Gapdh (sc-32233, Santa Cruz, United States), anti-Tubulin (AF5012, Beyotime, China), anti-Insulin Receptor β (IRβ) (ab69508, Abcam, United Kingdom), IRDye 800CW Goat anti-Rabbit IgG (P/N 926-32211, LI-COR Biotechnology, United States), and IRDye 800CW Goat anti-Mouse IgG (P/N 926-32210, LI-COR Biotechnology, United States).

### Knockdown of *Tfr1* and Modulation of Iron Levels in Adipocyte and Adipose Tissue

Immortalized beige and brown adipocytes were transfected with siRNA targeting *Tfr1* (si*Tfr1*) and a negative control siRNA (NC) with TransExcellentTM-siRNA (Cenji Biotechnology, Shanghai, China) at day 4 after differentiation. For iron level modulation, mature adipocytes were treated with 300 mM iron chelator deferoxamine (DFO) (D9533, Sigma, United States) or 160 μg/ml FeSO_4_ (F8633, Sigma, United States) for 48 h.

To silence *Tfr1* expression *in vivo*, si*Tfr1* or NC was transferred unilaterally into iWAT of *C57BL/6J* mice by a commercialized kit (Entranster-*in vivo*; Engreen Biosystem, Beijing, China) as previously reported (Zhou et al., 2015). The iWAT was taken 2 days later for further analysis. The experiment was approved by the Animal Care and the Animal Ethics Committee of East China Normal University.

### Immunostaining of Adipocytes and Adipose Tissues

For immunofluorescent staining, mature immortalized beige and brown adipocytes were fixed with 4% paraformaldehyde (PFA), blocked with 10% normal goat serum in 0.1% Tween-20/PBS, and incubated for 2 h with primary antibody Tfr1 (Abcam, ab84036) followed by AlexaFluor-488 goat anti-mouse IgG (B40941, Thermo Fisher, United States) as a secondary antibody. Slides were mounted with Vectashield (Vector Labs, United States) containing DAPI (P36931, Invitrogen, United States).

For immunohistochemistry staining, adipose tissues were placed in Bouin fixative, embedded in paraffin, and subsequently cut into 5-μm sections. Sections were deparaffinized, hydrated in xylene and graded ethanol, and rinsed in PBS for 5 min before incubated with pepsin (Sigma, United States). The sections were incubated for 10 min in 0.3% H_2_O_2_ to quench endogenous peroxidase activity. Sections were blocked and incubated at 4°C overnight with diluted polyclonal antibodies against Ucp1 (ab10983, Abcam, United Kingdom) and Tfr1 (ab84036, Abcam, United Kingdom) and with HRP-conjugated goat anti-rabbit IgG for 1 h. DAB chromogen (DAB Substrate Kit, H-2200, Vector Labs, United States) was used for peroxidase detection of immunoreactivity. Images were taken with a Zeiss 710 confocal microscope.

### MitoTracker Analysis

Mitochondria quality and membrane integrity were assessed by staining with MitoTracker^®^ Red CMXRos (Thermo Fisher, United States). Immortalized brown and beige adipocytes were differentiated to day 4 and transfected with si*Tfr1*. After 48 h, mature adipocytes were incubated with 100 nM MitotTracker for 20 min and then washed three times with PBS. Cells were fixed with 4% PFA for 10 min and fluorescent images were obtained using the Nikon inverted microscope ECLIPSE Ts2.

### Bioenergetic Analysis

Oxygen consumption rate (OCR) and extracellular acidification rate (ECAR) were measured using the XF24 Analyzers (Seahorse Bioscience). All compounds and media were prepared according to the manufacturers’ instructions. C3H10T1/2 cells were differentiated for 4 days and transfected with NC or si*Tfr1*; OCR and ECAR were measured 2 days later. OCR was measured under basal conditions, following the addition of ATP synthase inhibitor oligomycin, the mitochondrial uncoupler FCCP, and the complex III inhibitor antimycin A. ECAR was measured following the addition of glucose or 2-deoxyglucose (2-DG) at the indicated time.

### Determination of Lipid Peroxidation

Lipid peroxidation was assessed using lipid peroxidation kit (ab243377, Abcam, United Kingdom) according to the manufacturer’s instructions. Briefly, lipid peroxidation sensor was added to adipocytes and incubated for 30 min. After washing with HBSS, fluorescence was detected at Ex/Em = 490 nm (FITC)/530 nm (FITC) and 545 nm (TRITC)/600 nm (TRITC) with a fluorescence microscope. Adipocytes were photographed and the lipid peroxidation was quantified as the FITC/TRITC signal ratio (Molecular Devices). H_2_O_2_ treatment was used as a positive control.

### Detection of Intracellular ROS

Intracellular ROS levels were measured by the fluorescent 2′,7′-dichlorodihydrofluorescein (DCF) assay. Briefly, mature adipocytes were seeded in 96-well plates. After siRNA transfection for 48 h, culture medium was removed and adipocytes were incubated with 10 μmol/L DCFH-DA reagent (Reactive Oxygen Species Assay Kit, S0033S, Beyotime, China) for 20 min. After washing with serum-free cell culture medium to completely remove DCFH-DA, adipocytes were photographed and mean DCF fluorescent intensities were detected using a SpectraMax M2 microplate fluorometer (Molecular Devices).

### Determination of Iron Concentration

Intracellular iron level was determined using the iron assay kit (ab83366, Abcam, United Kingdom) according to the manufacturer’s instructions. Briefly, immortal brown and beige preadipocytes were differentiated following standard protocol to day 0, 2, 4, and 6. Cells were collected and washed in ice-cold PBS and homogenized in iron assay buffer on ice, followed by centrifugation (16,000 × *g*, 10 min) at 4°C to remove insoluble material. Supernatant was collected and incubated with iron reducer for 30 min at 25°C. Next, 100 μl of iron probe was added and mixed well with a horizontal shaker for 60 min at 25°C. The absorbance was then measured at 593 nm using SpectraMax 190 Microplate Reader (Molecular Devices).

### Cell Viability Assay

Cell viability was evaluated using the cell counting kit-8 (C0042, Beyotime, China). Briefly, 10 μl of CCK8 solution was added to each well and incubated for 2 h at 37°C. The optical density (OD) value of each well at 450 nm was measured and recorded using SpectraMax 190 Microplate Reader (Molecular Devices).

### Cellular TG and NEFA Measurement

After removing the cell culture medium, adipocytes were lysed with RIPA buffer and centrifuged to remove cell debris. Supernatant was collected from cellular TG (triglyceride) and NEFA (non-esterified free fatty acids) examination with Triglyceride Assay and NEFA Kit (A110-2-1, A042-2-1, Jincheng, Nanjing, China) and normalization to total protein concentration.

### Lentiviral Constructions and Infection

Transferrin receptor lentiviral particles were constructed and concentrated by GeneChem company (Shanghai). Immortalized beige preadipocytes were infected with 2 × 10^9^ PFU lentiviral particles carrying Tfr1 or Gfp with 4 mg/ml sequa-brene (S2667, Sigma, United States) for 24 h and switched to fresh medium for another 2 days. Samples were collected for real-time PCR and Western blot analysis.

### Statistical Analysis

Data are presented as means ± SEM. Student’s *t* test analysis was performed with GraphPad Prism software and *P* < 0.05 was considered as significant.

## Results

### *In silico* Analysis of Cell Surface Molecules of Beige Versus White Adipocytes

**FIGURE 1:**
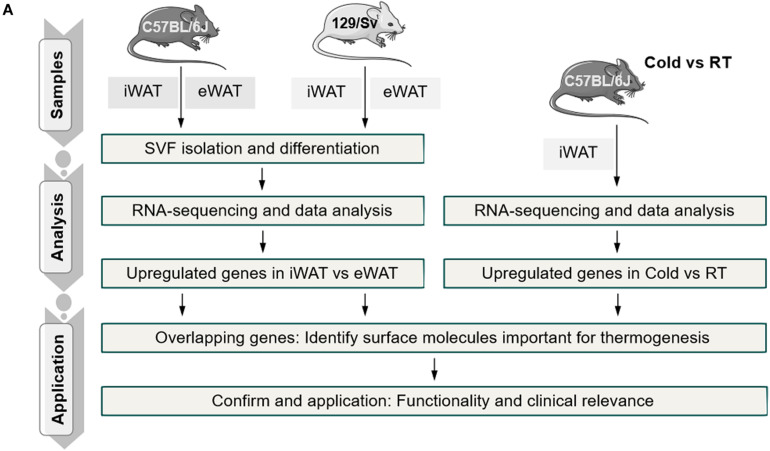
Schematic representation of the experimental workflow.

**FIGURE 2:**
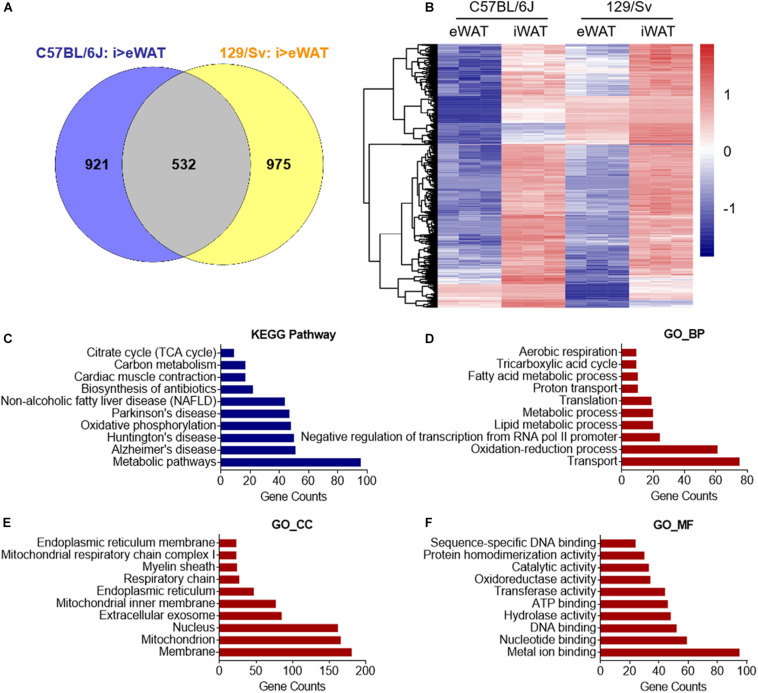
*In silico* analysis of cell surface molecule of beige versus white adipocyte. **(A,B)** Genes featured high expression in primary beige adipocytes from iWAT versus primary white adipocytes from eWAT (fold change >2 and *P* < 0.05) of C57BL/6J and 129/Sv mice were overlapped and shown in **(A)** a Venn diagram. **(B)** Cluster analysis of overlapped genes that showed high expressions in beige vs. white adipocytes in C57BL/6J and 129/Sv mice. Upregulation and downregulation of genes were shown in red and blue, respectively. **(C)** KEGG pathway enrichment analysis of overlapped genes that showed high expressions in beige compared to white adipocytes in C57BL/6J and 129/Sv mice with top 10 different gene counts. **(D–F)** GO analysis of overlapped genes that showed high expressions in beige compared to white adipocytes in C57BL/6J and 129/Sv mice. Top 10 GO terms in biological process (BP) **(D)**, cellular component (CC) **(E)**, and molecular function (MF) **(F)** were presented. SVFs, stromal vascular fractions; iWAT, inguinal adipose tissue; eWAT, epididymal adipose tissue; RT, room temperature.

Primary SVFs were isolated from iWAT, which undergoes the most profound induction of beige adipocytes, and from eWAT, which are particularly resistant to beigeing and are considered white adipocytes from 2-month-old *C57BL/6J* and *129/Sv* male mice, the two most commonly used inbred mice in scientific studies. These SVFs were cultured and induced for differentiation with brown gene program-inducing cocktails (Tseng et al., 2008). After 8 days, fully differentiated mature adipocytes of iWAT and eWAT origin were subjected to RNA-sequencing analysis (Figure 1). To identify thermogenic-specific genes, we focused on oscillating genes that feature significant changes (>2-fold change and *P* < 0.05) in iWAT versus eWAT in both *C57BL/6J* and 129/Sv mice, which rendered 532 genes after overlapping (Figures 2A,B). KEGG pathway enrichment analysis revealed that these genes were enriched for metabolic pathways (Figure 2C). Notably, top terms in GO analysis of BP, CC, and MF are enriched for transport, membrane, and metal ion binding, respectively (Figures 2D–F). Furthermore, since cold stimuli strongly induce browning of beige adipocytes, we thus performed RNA-sequencing using iWATs from *C57BL/6J* mice maintained under room temperature or under 1-week cold stimuli. KEGG as well as GO analysis on oscillating genes of iWAT under cold or room temperature with significant changes (>2-fold change and *P* < 0.05) also enriched similar results, particularly in metal ion binding/transport signaling (Supplementary Figures S1A–D). These results suggested a potential critical role of metal ion binding/transporting membrane proteins in beige fat function.

### Tfr1 Marks Thermogenic Adipocyte Both *in vivo* and *in vitro*

**FIGURE 3:**
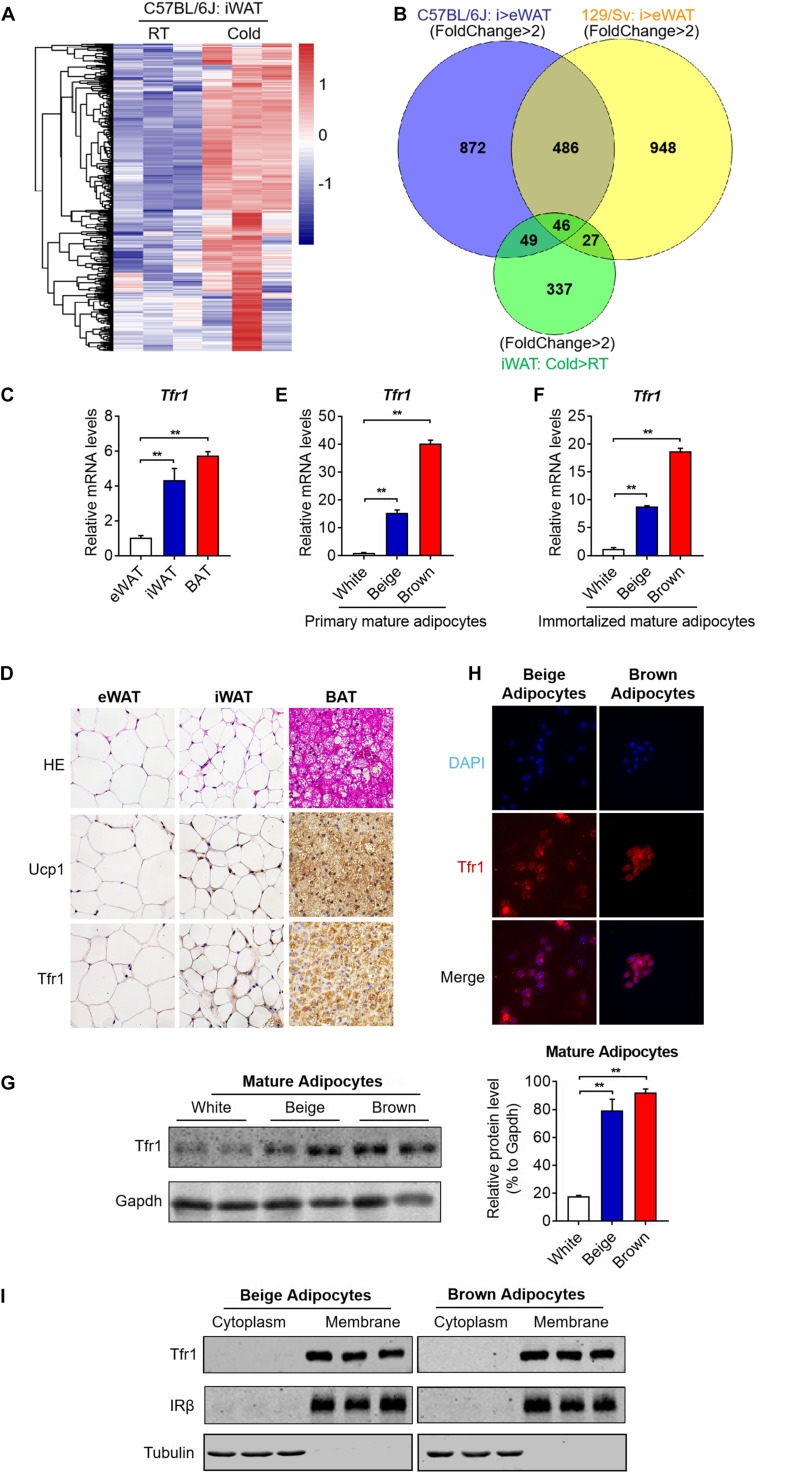
Screening by *silico* analysis and validation of the thermogenic adipocyte surface marker. **(A)** Cluster analysis of overlapped genes that showed high expressions in iWAT from C57BL/6J mice under cold stimuli vs. RT (fold change >2 and *P* < 0.05). Upregulation and downregulation of genes were shown in red and blue, respectively. **(B)** Genes that featured high expressions in beige compared to white adipocytes in C57BL/6J and 129/Sv mice as well as genes that showed high expressions in iWAT from C57BL/6J mice under cold stimuli vs. RT (fold change >2 and *P* < 0.05) were overlapped and shown in a Venn diagram. **(C)** Gene expression analysis of *Tfr1* in eWAT, iWAT, and BAT. **(D)** Representative H&E staining and immunostaining of Ucp1 and Tfr1 of eWAT, iWAT, and BAT. **(E,F)** Gene expression analysis of *Tfr1* in primary **(E)** and immortalized **(F)** mature adipocytes. **(G)** Western blot analysis (left) and quantification (right) of Tfr1 in thermogenic and white adipocytes. **(H)** Representative pictures of immunofluorescent staining of Tfr1 in thermogenic adipocytes. Tfr1 and nucleus were indicated in red and blue, respectively. **(I)** Western blot analysis of Tfr1 in cytoplasm and membrane fractions of immortalized thermogenic adipocytes using the membrane protein IRβ and cytosolic protein Tubulin as positive controls. Data are presented as mean ± SEM. iWAT, inguinal adipose tissue; eWAT, epididymal adipose tissue; BAT, brown adipose tissue. **P < 0.01. The results are representative of at least three independent experiments.

To identify surface molecules important for thermogenesis, we overlapped enriched gene sets from *in vitro* induced beige adipocytes versus white adipocytes derived from *C57BL/6J* mice and 129/Sv mice, as well as *in vivo* beige adipocytes under cold stimuli versus room temperature (Figure 3A). Interestingly, this overlap generated 46 genes that characterize both specific expression in beige adipocytes versus white adipocytes and strong induction upon cold stimuli, indicative of their potential function in the browning process (Figure 3B and Supplementary Table S2). Of note, among the list, Tfr1 (also named as Tfrc), a receptor specialized for cellular iron uptake, is the only gene encoding a membrane protein. Thus, we next focused our following studies on Tfr1.

We first validated Tfr1 expression levels in different adipose depots. Consistent with RNA-seq results, we found that *Tfr1* mRNA is highly expressed in mice iWAT and BAT, far eclipsed that in eWAT, suggesting the specific expression of *Tfr1* in thermogenic adipose tissues (Figure 3C). Immunohistological analysis showed that Tfr1 protein also featured dominant expressions in iWAT and BAT as compared to eWAT in a pattern that resembled Ucp1 expression (Figure 3D). Furthermore, profound Tfr1 mRNA and proteins also existed in primary or immortalized mature beige and brown adipocytes versus white adipocytes (Figures 3E–G). We found that Tfr1 characterizes cell membrane localization in beige and brown adipocytes as shown by immunostaining and Western blot using a classic cell surface receptor IRβ and a cytosolic protein Tubulin as positive controls (Figures 3H,I and Supplementary Figure S2A). Of note, high-resolution confocal images suggested that a small fraction of Tfr1 also underwent internalization (Supplementary Figure S2A), as previously reported (Jabara et al., 2016).

Meanwhile, it has to be noted that, by generating a cDNA tissue library containing brain, heart, liver, spleen, lung, kidney, pancreas, gastrocnemius, and soleus muscle from 2-month-old *C57BL/6J* male mice, we found that Tfr1 also expressed in other tissues, overall suggesting that Tfr1 may be critical for iron homeostasis in various tissues (Supplementary Figure S2B). Though it may not serve as a unique marker for brown/beige adipocytes, Tfr1 may be valuable to distinguish between thermogenic versus white adipocytes.

### Tfr1 Predominantly Expressed in Mature Thermogenic Adipocytes

**FIGURE 4:**
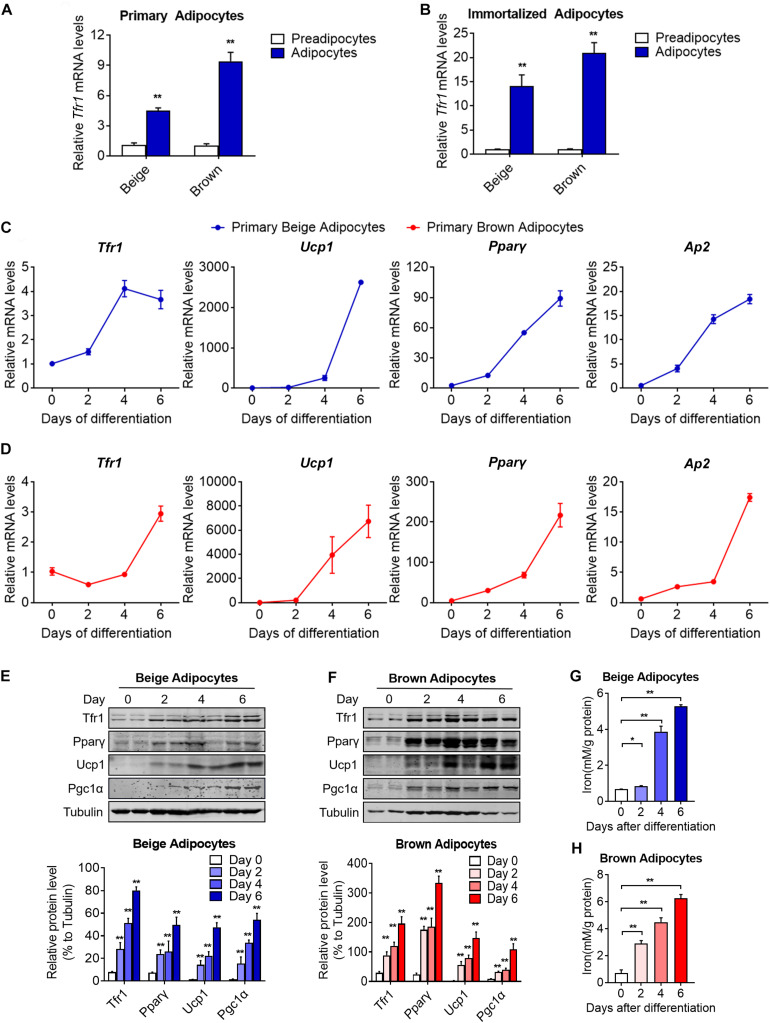
Tfr1 expression levels and iron concentrations during thermogenic adipocytes differentiation. **(A,B)** Gene expression analysis of *Tfr1* in mature primary **(A)** and immortalized **(B)** thermogenic adipocytes compared to preadipocytes. **(C,D)** Gene expression analysis of *Tfr1*, *Ucp1*, *Ppar*γ, and *Ap2* at indicated time points during differentiation in primary beige **(C)** and brown **(D)** adipocytes. **(E,F)** Western blot analysis (up) and quantification (down) of Tfr1, Pparγ, Ucp1, and Pgc1α at indicated time points during differentiation in immortalized beige **(E)** and brown **(F)** adipocytes. **(G,H)** Intracellular iron level at indicated time points during differentiation in beige **(G)** and brown **(H)** adipocytes. Data are presented as mean ± SEM. *P < 0.05; **P < 0.01. The results are representative of at least three independent experiments.

Beige and brown preadipocytes differentiate into mature adipocytes to become fully functional metabolically and thermogenically. The expression levels of an array of genes important for thermogenic adipocyte functions are induced gradually during the differentiation process and peaked in mature adipocytes, i.e., Pparγ, aP2, and Ucp1. We found that *Tfr1* expressed predominantly in mature adipocytes compared to preadipocytes in SVFs from mouse iWAT and BAT and immortalized beige and brown adipocytes (Figures 4A,B). Moreover, Tfr1 expressions increased during the differentiation process in a pattern similar to Pparγ, aP2, and Ucp1 (Figures 4C–F and Supplementary Figure S3), accompanied by increased cellular iron levels (Figures 4G,H). Of note, *Tfr1* knockdown in adipocytes during differentiation affected gene programs related to adipogenesis, lipid synthesis, oxidative phosphorylation, fatty acid oxidation, as well as cellular TG and NEFA levels (Supplementary Figure S4). These results suggested that Tfr1 may play biological roles mainly in mature thermogenic adipocytes.

### Tfr1 Levels in Thermogenic Adipocytes Responds to Metabolic Status

**FIGURE 5:**
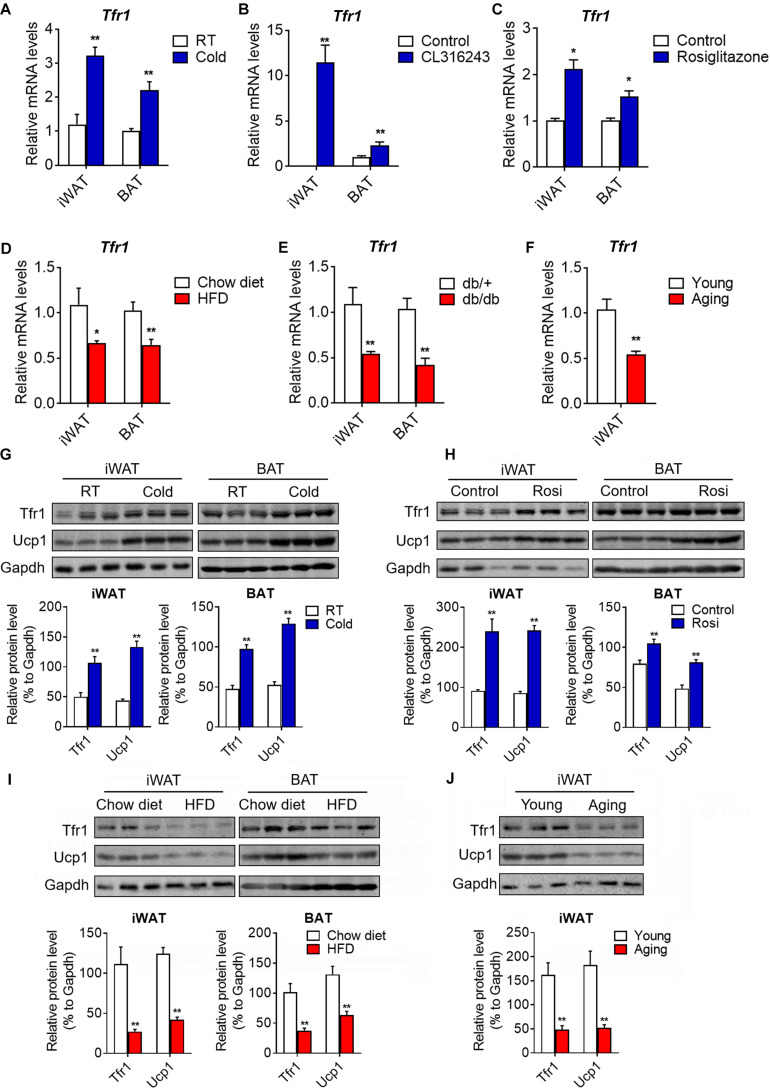
Tfr1 expression upon physiological and pathological stimulation *in vivo* and *in vitro*. **(A–C)** Gene expression analysis of *Tfr1* in BAT and iWAT from mice under chronic cold condition **(A)**, CL316243 injection **(B)**, or Rosiglitazone treatment **(C)**, compared to their individual control mice. **(D–F)** Gene expression analysis of *Tfr1* in BAT and iWAT from HFD-fed **(D)**, *db/db* **(E)**, or aging **(F)** mice, compared to their individual control mice (Chow diet, db/+ or young mice). **(G,H)** Western blot analysis (up) and quantification (down) of Tfr1 and Ucp1 in BAT and iWAT from mice under chronic cold condition **(G)** or Rosiglitazone treatment **(H)**, compared to their control mice. **(I,J)** Western blot analysis (up) and quantification (down) of Tfr1 and Ucp1 in BAT and iWAT from HFD-fed **(I)** or aging **(J)** mice, compared to their control mice. Data are presented as mean ± SEM. iWAT, inguinal adipose tissue; BAT, brown adipose tissue; Rosi: Rosiglitazone. *P < 0.05; **P < 0.01. *n* = 5 for each group.

**FIGURE 6:**
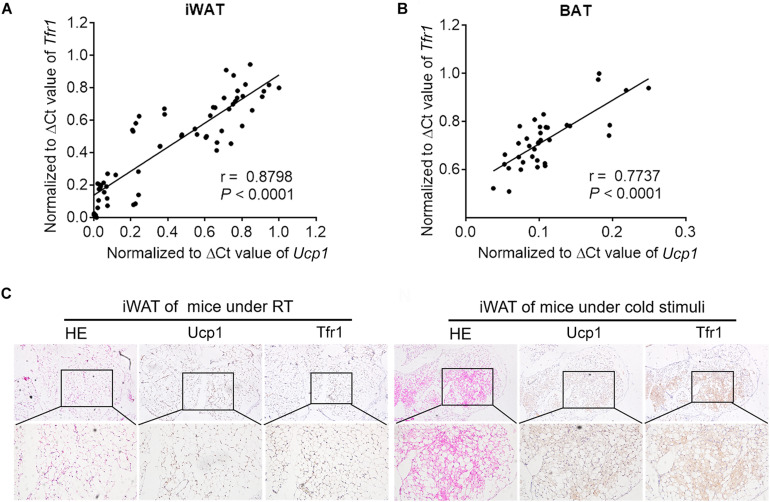
The correlation between Tfr1 and Ucp1. **(A,B)** The correlation between Tfr1 and Ucp1 mRNA levels in iWAT **(A)** and BAT **(B)**. ΔCt values were used for analysis. **(C)** Representative H&E staining and immunostaining of Ucp1 and Tfr1 of iWAT from mice with or without cold treatment. iWAT, inguinal adipose tissue; BAT, brown adipose tissue. *n* = 5 for each group.

To further understand the role of Tfr1 in thermogenic adipocytes, we examined Tfr1 levels in fat tissues under various physiological or pathological scenarios that are known to induce or suppress beige/brown fat thermogenic capacity. Importantly, compared to mice housed at room temperature, *Tfr1* levels were highly induced along with *Ucp1*, a marker for thermogenic activation, in beige and brown fat from mice maintained in cold environment for 1 week (Figure 5A and Supplementary Figure S5A). β3-adrenergic receptor agonist CL316243 and Pparγ full agonist Rosiglitazone treatment have been shown to promote white fat browning in mice (Ohno et al., 2012; De Jong et al., 2015). Consistently, daily CL316243 injection for 2 weeks and Rosiglitazone injection for 10 days in mice both significantly elevated *Tfr1* and *Ucp1* expressions in iWAT and BAT (Figures 5B,C and Supplementary Figures S5B,C). On the other hand, we tested *Tfr1* levels in beige and brown fat from multiple mice models with impaired thermogenesis and increased whitening phenotype, including diet-induced obese mice (HFD mice), genetic obese mice (*db/db* mice), as well as aging mice. We found significantly decreased *Tfr1* levels along with declined *Ucp1* expressions compared to control mice (Figures 5D–F and Supplementary Figures S5D–F). Moreover, protein levels of Tfr1 in iWAT and BAT changed consistently with its mRNA levels (Figures 5G–J). Importantly, *Tfr1* levels were positively correlated with the expressions of *Ucp1* in iWAT and BAT (Figures 6A,B). In addition, Tfr1 and Ucp1 proteins also showed similar high induction in iWAT under cold treatment (Figure 6C), suggesting a tight correlation of Tfr1 levels with the metabolic status of thermogenic adipocytes. Furthermore, the correlation of *Tfr1* expression with *Ucp1* was recapitulated in immortalized brown/beige adipocytes treated with Forskolin or H89, the classic *in vitro* cellular models of thermogenic activation and suppression (Supplementary Figures S5G–J). Meanwhile, in addition to *Tfr1* and *Ucp1*, iron metabolic regulator *Irp1* and *Irp2* levels were also increased in iWAT upon cold stimulation compared to thermoneutral environment, suggesting a systematic mobilization of iron regulatory genes (Supplementary Figures S5K,L). Overall, these results indicated that Tfr1 levels fluctuated in response to physiological and pathological changes impacting thermogenic adipocyte functions both *in vivo* and *in vitro*.

### Tfr1 Is Indispensable for Functional Thermogenic Adipocytes

**FIGURE 7:**
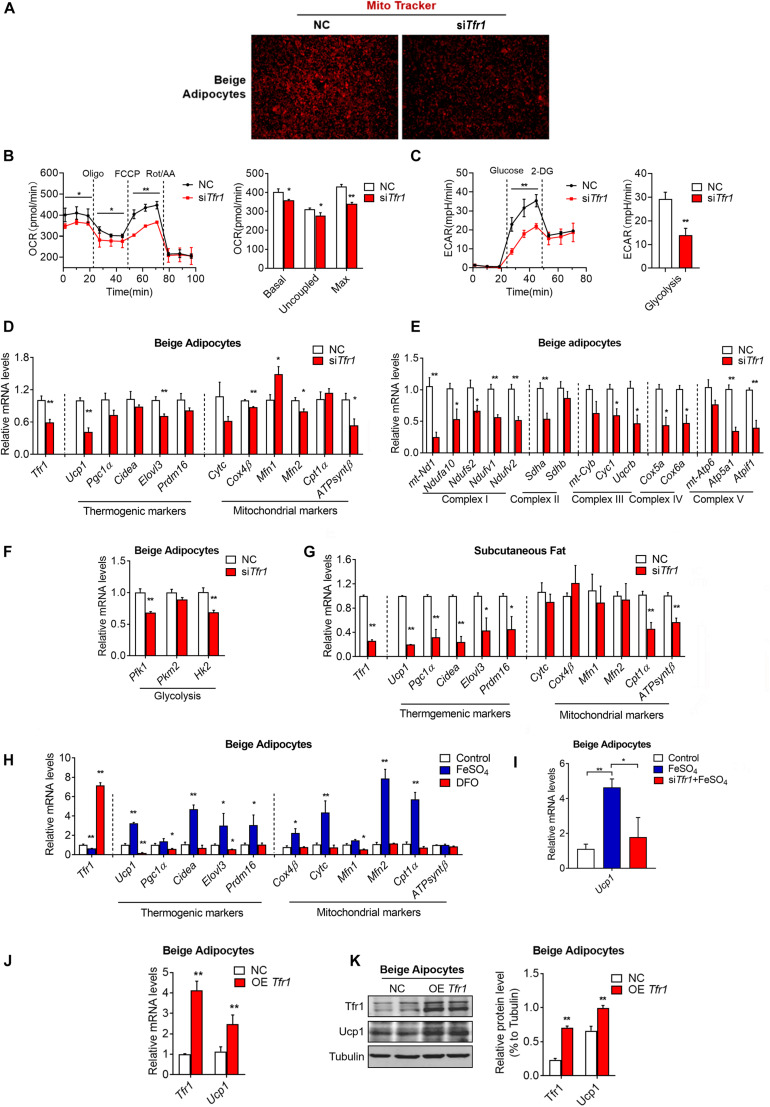
The effect of *Tfr1* knockdown (si*Tfr1*) on thermogenic adipocyte function *in vivo* and *in vitro*. **(A)** Mito Tracker staining of mature beige adipocytes with or without *Tfr1* knockdown (si*Tfr1*). **(B,C)** Measurement of cellular oxygen consumption rate (OCR) and ECAR of mature C3H10T1/2 adipocytes with or without *Tfr1* knockdown (si*Tfr1*). **(D–F)** Gene expression analysis of *Tfr1*, thermogenic markers, mitochondrial markers **(D)**, mitochondrial respiratory chain complex markers **(E)**, and glycolytic markers **(F)** from beige adipocytes with or without *Tfr1* knockdown (si*Tfr1*). **(G)** Gene expression analysis of *Tfr1*, thermogenic markers, and mitochondrial markers from subcutaneous fat with or without *Tfr1* knockdown (si*Tfr1*). **(H)** Gene expression analysis of *Tfr1*, thermogenic markers, and mitochondrial markers from DFO-treated or FeSO_4_-treated beige adipocytes. **(I)** Ucp1 mRNA levels in beige adipocytes treated with FeSO_4_ with or without *Tfr1* knockdown (si*Tfr1*). **(J)** Gene expression analysis of *Tfr1* and *Ucp1* from beige adipocytes after Tfr1 overexpression (OE Tfr1). **(K)** Western blot analysis (left) and quantification (right) of Tfr1 and Ucp1 from beige adipocytes after Tfr1 overexpression (OE Tfr1). Data are presented as mean ± SEM. *P < 0.05; **P < 0.01. The results are representative of at least three independent experiments. OCR, oxygen consumption rate; ECAR, extracellular acidification rate; NC, negative control; DFO, deferoxamine.

Since we have demonstrated that Tfr1 is highly expressed in thermogenic adipocytes and responds to metabolic changes, we next examined whether Tfr1 plays active roles in mature thermogenic adipocyte functionality. Indeed, *Tfr1* suppression in mature brown and beige adipocytes resulted in impaired mitochondrial quality and membrane integrity as revealed by MitoTracker staining (Figure 7A and Supplementary Figure S6A). Moreover, *Tfr1* knockdown suppressed cellular oxygen consumption and glycolysis as revealed by Seahorse analysis (Figures 7B,C), which was consistent with reduced expressions of brown gene programs, including thermogenic genes (*Ucp1*, *Pgc1a*, *Cidea*, *Elovl3*, and *Prdm16*), mitochondrial genes (*CytC*, *Cox4*β, *Mfn1*, *Mfn2*, *Cpt1a*, and *ATPsynt*β), mitochondrial respiratory complex genes (I–V), and glycolytic genes (*Pfk1*, *Pkm2*, and *Hk2*) both *in vitro* and *in vivo* (Figures 7D–G and Supplementary Figures S6B–D). These data suggested that Tfr1 might be indispensable for energy expenditure and mitochondrial function in thermogenic adipocytes.

Transferrin receptor exerts its function mainly through binding to transferrin for extracellular iron uptake. Iron has been shown to be vital in a plethora of cellular processes. For example, iron plays a critical role in oxidative phosphorylation by functioning as a metal core of iron–sulfur (Fe–S) complex and promoting electron transfer by shifting from Fe^2+^ to Fe^3+^ states in respiratory chain complexes I, II, and III located in the inner mitochondrial membrane (Rouault and Tong, 2005). We thus examined whether iron homeostasis is critical for thermogenic functions of mature beige adipocytes. Intriguingly, clearance of cellular iron concentration through treatment of the iron chelator DFO decreased expressions of brown gene programs, while forced elevation of iron levels by addition of FeSO_4_-induced brown gene programs in thermogenic adipocytes (Figure 7H). Importantly, *Tfr1* knockdown largely blunted the induction of *Ucp1* by FeSO_4_, indicating that iron may enhance thermogenesis through Tfr1 (Figure 7I). On the other hand, overexpression *Tfr1* in beige adipocytes increased mRNA and protein levels of thermogenic marker Ucp1 (Figures 7J,K). Thus, these results suggested that iron metabolism may be important for energy homeostasis in thermogenic fat, possibly through its membrane transporter, Tfr1.

**FIGURE 8:**
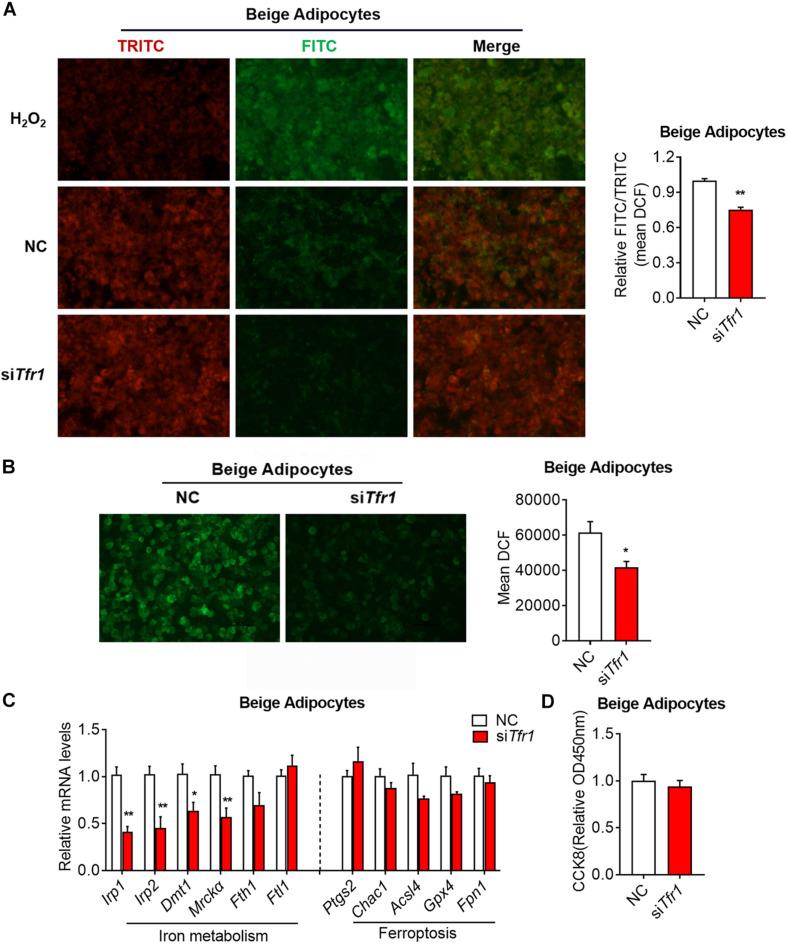
The effect of *Tfr1* knockdown (si*Tfr1*) on cellular stress and iron metabolism in beige adipocytes. **(A)** Lipid peroxidation analysis with pre-lipid peroxidation (red, TRITC) and post-lipid peroxidation (green, FITC) from beige adipocytes after *Tfr1* knockdown (si*Tfr1*). Left: representative images of lipid peroxidation staining; right: lipid peroxidation fluorescence intensity presented as FITC/TRITC signal ratio. **(B)** ROS activity analysis with DCF (green) from beige adipocytes after *Tfr1* knockdown (si*Tfr1*). Left: representative images of ROS staining; right: ROS fluorescence intensity. **(C)** Gene expression analysis of iron metabolic and ferroptotic markers from beige adipocytes with or without *Tfr1* knockdown (si*Tfr1*). **(D)** Relative cellular viability analysis from beige adipocytes with or without *Tfr1* knockdown (si*Tfr1*). Data are presented as mean ± SEM. *P < 0.05; **P < 0.01. The results are representative of at least three independent experiments. ROS, reactive oxygen species; NC, negative control.

We have shown that *Tfr1* knockdown disrupted mitochondrial integrity and functionality, and we further assessed whether it is caused by possible cell death. In fact, we showed that *Tfr1* knockdown reduced cellular lipid peroxidation and cellular ROS levels in brown and beige adipocytes (Figures 8A,B and Supplementary Figures S7A,B). Besides, although iron metabolic gene levels were suppressed upon *Tfr1* knockdown, ferroptotic gene levels were unaltered (Figure 8C and Supplementary Figure S7C). Furthermore, the cell viability assay suggested that *Tfr1* knockdown did not lead to ferroptosis-induced cell death (Figure 8D and Supplementary Figure S7D).

## Discussion

In the present study, we identified Tfr1, previously named CD71, as a surface molecule that features specific expression in thermogenic adipocytes, including brown and beige adipocytes, versus white adipocytes. These data showed that *Tfr1* deficiency reduced gene programs of iron uptake and storage, thermogenesis, and mitochondrial functionality. Furthermore, we showed that the absence of *Tfr1* impaired cellular energetics and glycolysis as evaluated by cellular oxygen consumption (OCR) and ECAR analysis using Seahorse instruments, indicative of a vital role of Tfr1/iron metabolism on cellular energy homeostasis. On the other hand, we found that deteriorating events such as lipid peroxidation and cellular ROS stress were suppressed after *Tfr1* knockdown, without obvious effects on ferroptosis or cell viability, suggesting a multifaceted impact of Tfr1 in thermogenic adipocytes.

A number of studies have provided potential molecules that specifically expressed in brown and beige adipocytes versus white adipocytes for better target efficiency. For example, by characterizing single cell clones from isolated preadipocyte of white, brown, and beige fat, it has been shown that compared to white adipocytes, brown adipocytes express high levels of Ucp1, Zic1, and Lhx8, and beige adipocytes express Tbx1, Tmem26, and Tnsfrsf9, in addition to Ucp1 (De Jong et al., 2015). However, utilization of these molecules as therapeutic targets encountered difficulties since they are either intracellular markers that are not suitable for direct targeting *in vivo*, or under debate for expression specificity (Xue et al., 2015). On the other hand, by data mining gene expression database, Ussar et al. (2014) searched surface markers that feature high expression association with Ucp1, the critical thermogenic marker gene. They identified surface proteins Pat2 and P2rx5 that show high specificity to brown/beige adipocytes, though whether Pat2 and P2rx5 affect thermogenic adipocyte functions remained unexamined. In the present study, we proposed Tfr1 as a previously unidentified cell surface molecule for thermogenic adipocytes with functional assessment. Tfr1 is highly responsive to environmental cues, and its levels were induced upon classical browning stimuli, including cold, β3 adrenergic signaling, and Pparγ agonist treatment, while its levels negatively responded to whitening stimuli, including HFD and aging, suggesting that Tfr1 levels may be a suitable indicator that dynamically reflects the amount and function of thermogenic adipocytes under various physiological status. Moreover, *Tfr1* knockdown in mature beige adipocytes led to reduced mitochondrial integrity and reduced brown gene programs, indicative of an active role of Tfr1 in beige adipocyte function. Interestingly, comparing Tfr1 with previously reported markers revealed a difference in their responses to browning stimuli. For example, Tmem26 levels did not change under cold exposure or β3 adrenergic stimulation with CL316243. Pat2 levels were induced by CL316243 induction but not cold stimulation, while Tfr1 and P2rx5 sensed both stimulations. Thus, it would be informative to study the detailed functions of these intracellular or surface markers of thermogenic adipocytes.

Of note, we did not identify cell surface markers that show differential expression pattern between brown and beige adipocytes. Similarly, Ussar et al. (2014) demonstrated that Pat2 and P2rx5 were specific surface markers for both brown/beige adipocyte. It is possible that though beige and brown adipocytes have distinct developmental origins, they share high similarity in functions after activation and thus may have similar spectrum of surface markers. Besides, we found that, although Tfr1 is predominantly expressed in mature thermogenic adipocytes, it is also detectable in other tissues, not unlike that of Pat2/P2rx5. Given the specificity and membrane location feature, Tfr1 would be an ideal surface marker with local applications for identifying and sorting thermogenic adipocytes in isolated mice white fat or in human fat tissues.

Transferrin receptor plays an important role for cellular iron uptake and homeostasis (Gammella et al., 2017). Recently, via analyzing Tfr1 fat-conditional knockout mice, Tfr1 was found to be vital for fat biology since Tfr1 deficiency promoted the transdifferentiation of brown adipocytes into muscle-like cells, blunted cold-induced browning of white fat and thermogenesis, but led to HFD-induced hyperlipidemia, insulin resistance, and local inflammation (Li et al., 2020). The present study and the study of Li et al. (2020) share a few similar conclusions, but the present study also provided novel insights into and mechanistic details about Tfr1 and iron metabolism in thermogenic adipocytes. Firstly, beige adipocytes share many similarities with white adipocytes and are hard to distinguish and target, which limited their implications. Moreover, it has been shown that physiological stimuli like cold induced different responses in beige adipocytes compared to pharmacologic stimuli. Thus, the fundamental goals of the present study are to identify a specific surface marker that plays important roles under a physiological setting while, at the same time, distinguishing beige and white adipocytes. To achieve this, we performed unbiased RNA-seq in beige and white fat depots from two mouse stains of different genetic backgrounds, as well as beige fat from mice with or without cold acclimation, and overlapped differential expressions of surface molecules from these datasets to find candidates that have tight physiological association with thermogenic ability and distinct expression in beige versus white fat. In comparison, Li et al. used beige fats from control mice and mice with pharmacologic stimulation for β-adrenergic activation for RNA-seq analysis. Secondly, we provided additional mechanistic insights including cellular oxygen consumption and glycolysis measured by Seahorse instrument, lipid peroxidation and cellular ROS levels, and Tfr1 gain-of-function analysis, further suggesting the importance of iron homeostasis in fat biology. Lastly, Li et al. (2020) studied Tfr1 functionality through adipocyte developmental/differentiation stages, including differentiation of primary SVF isolated from wild-type and Tfr1 fat-conditional knockout mice, as well as C3H10T1/2 cells stably transfected with or without Tfr1 shRNA. On the other hand, we focused on the role of Tfr1 in mature beige adipocytes by utilizing a siRNA system to manipulate Tfr1 levels directly in differentiated adipocytes. Thus, our study together with Li’s study provided a comprehensive picture of Tfr1 function in various stages of thermogenic adipocytes.

In addition to iron, other metals, such as copper (Cu), calcium, and zinc, also play fundamental roles in mitochondrial functionality and are involved in fat biology. Specifically, complexes I–IV of the electron transport chain in mitochondria require Fe–S clusters and Cu centers for normal functionality (Maio et al., 2017; Boulet et al., 2018). Besides, calcium is one of the most well-studied ions in mitochondria. An increase in mitochondrial calcium levels can activate ATP production, brown fat thermogenesis, and browning of white fat by altering the activity of calcium-sensitive proteins such as NCLX and SERCA2b (Williams et al., 2015; Ding et al., 2018; Pathak and Trebak, 2020). Zinc may also play important roles in mitochondrial functionality by serving as ion center for zinc finger proteins. Zinc finger protein family is one of the largest protein family. Various critical zinc finger proteins, including Prdm16, Znf638, Zfp423, etc., have been shown to play critical roles in mitochondrial and fat biology (Shao et al., 2016a,b; Kissig et al., 2017; Perie et al., 2019). Thus, further studies are warranted to understand the finely tuned functions of ion signaling in mitochondrial functionality and thermogenesis.

In addition to the vital function of Tfr1 on mature thermogenic adipocytes, we also assessed possible involvement of Tfr1 in beige progenitor cells. Interestingly, based on a recent study (Nicole et al., 2020, Nature), we analyzed Tfr1 levels from SVF of beige adipocytes during aging using data from Mouse Aging Cell Atlas. Interestingly, the numbers of Tfr1-positive SVFs declined during the aging process (Supplementary Figure S8), suggesting the involvement of iron metabolism in functionality of progenitor cells and cellular senescence.

In summary, we identified that Tfr1 is expressed predominantly in thermogenic adipocytes versus white adipocyte, and its expression levels are tightly correlated with browning status. Importantly, Tfr1 is a functional cell surface marker as its deficiency led to impaired mitochondrial quality and membrane integrity in thermogenic adipocytes, overall suggesting that Tfr1 gene may serve as a functional surface molecule of thermogenic adipocytes for future application purposes.

## Data Availability Statement

The authors acknowledge that the data presented in this study must be deposited and made publicly available in an acceptable repository, prior to publication. Frontiers cannot accept a manuscript that does not adhere to our open data policies.

## Ethics Statement

The animal study was reviewed and approved by Shanghai Jiao Tong University (Shanghai, China), East China Normal University (Shanghai, China).

## Author Contributions

LX, JW, XM, and XG conceived the project and designed the experiments. JQ, SW, ZZ, and YC carried out most of the experiments. CL, DW, JS, XC, LM, and MN assisted in some experiments and data analysis. SX, JY, TH, and YH provided rodent biological samples for association analysis. XM, JW, and LX wrote the manuscript. XG contributed valuable comments and advice on the manuscript. All authors contributed to the article and approved the submitted version.

## Conflict of Interest

The authors declare that the research was conducted in the absence of any commercial or financial relationships that could be construed as a potential conflict of interest.

## Footnotes

1 http://bioinformatics.psb.ugent.be/webtools/Venn/

2 https://david.ncifcrf.gov/

## Supplementary Material

Supplementary Figure 1 *In silico* analysis of significantly altered genes in iWAT of mice with or without cold exposure. **(A–D)** KEGG pathway enrichment analysis **(A)** and GO analysis **(B–D)** of significantly altered genes in iWAT from mice under cold exposure or room temperature with top 10 different gene counts.

Supplementary Figure 2 Tfr1 expression pattern in multiple tissues. **(A)** Confocal images of immunofluorescence staining of Tfr1 (Phalloidin-Alexa488, green) and DAPI (blue). **(B)** Gene expression analysis of *Tfr1* in heart, liver, spleen, lung, kidney, brain, pancreas, gastrocnemius, and soleus muscle of 2-month-old C57BL/6J mice.

Supplementary Figure 3 Tfr1 expression levels during immortalized adipocyte differentiation. **(A–C)** Gene expression analysis of *Tfr1*, *Ucp1*, *Ppar*γ, and *Ap2* at indicated time point during differentiation in immortalized beige adipocytes **(A)**, C3H10T1/2 adipocytes **(B)**, and immortalized brown adipocytes **(C)**. Data are presented as mean ± SEM. ^∗^*P* < 0.05; ^∗∗^*P* < 0.01. The results are representative of at least three independent experiments.

Supplementary Figure 4 The effect of *Tfr1* knockdown (si*Tfr1*) on lipid metabolism in thermogenic adipocytes. **(A,B)** Gene expression analysis of *Tfr1*, adipogenesis markers, lipid synthesis markers, oxidative phosphorylation markers and fatty acid oxidation markers from beige **(A)** and brown **(B)** adipocytes after 2 days of *Tfr1* knockdown (si*Tfr1*) on day 2 of differentiation. **(C)** Gene expression analysis of *Tfr1*, adipogenesis markers, lipid synthesis markers, oxidative phosphorylation markers, and fatty acid oxidation markers from beige adipocytes after 2 days of *Tfr1* knockdown (si*Tfr1*) on day 4 of differentiation. **(D)** TG and NEFA contents from beige adipocytes after 2 days of *Tfr1* knockdown (si*Tfr1*) on day 4 of differentiation. **(E)** Gene expression analysis of *Tfr1*, adipogenesis markers, lipid synthesis markers, oxidative phosphorylation markers, and fatty acid oxidation markers from brown adipocytes after 2 days of *Tfr1* knockdown (si*Tfr1*) on day 4 of differentiation. **(F)** TG and NEFA contents from brown adipocytes after 2 days of *Tfr1* knockdown (si*Tfr1*) on day 4 of differentiation. Data are presented as mean ± SEM. ^∗^*P* < 0.05; ^∗∗^*P* < 0.01. The results are representative of at least three independent experiments. NC, negative control.

Supplementary Figure 5 Tfr1 expression level in iWAT and BAT under physiological and pathological stimuli. **(A–C)** *Ucp1* mRNA levels in iWAT and BAT from mice under chronic cold condition **(A)**, CL316243 injection **(B)** or Rosiglitazone treatment **(C)**, compared to their individual control mice. **(D–F)** *Ucp1* mRNA levels in iWAT and BAT from HFD diet fed **(D)**, *db/db* **(E)** or aging **(F)** mice, compared to their individual control mice (Chow diet, db/+ or young mice). **(G–J)** *Ucp1* **(G,H)** and *Tfr1* **(I,J)** mRNA levels in immortalized adipocytes treated with or without Forskolin (FSK) and H89. **(K–L)** *Irp1*, *Irp2* **(K)**, *Tfr1* and *Ucp1* **(L)** mRNA levels in iWAT from mice under chronic cold condition, compared to thermalneutral condition. Data are presented as mean ± SEM. iWAT, inguinal adipose tissue; BAT, brown adipose tissue; RT, room temperature. ^∗^*P* < 0.05; ^∗∗^*P* < 0.01. The results are representative of at least three independent experiments.

Supplementary Figure 6 The effect of *Tfr1* knockdown (si*Tfr1*) on mitochondrial function in brown adipocytes. **(A)** Mito Tracker staining of mature brown adipocytes with or without *Tfr1* knockdown (si*Tfr1*). **(B)** Gene expression analysis of *Tfr1*, thermogenic markers and mitochondrial markers from brown adipocytes with or without *Tfr1* knockdown (si*Tfr1*). **(C)** Gene expression analysis of five mitochondrial respiratory chain complex markers from brown adipocytes with or without *Tfr1* knockdown (si*Tfr1*). **(D)** Gene expression analysis of glycolysis markers from brown adipocytes with or without *Tfr1* knockdown (si*Tfr1*). Data are presented as mean ± SEM. ^∗^*P* < 0.05; ^∗∗^*P* < 0.01. The results are representative of at least three independent experiments. NC, negative control.

Supplementary Figure 7 The effect of *Tfr1* knockdown (si*Tfr1*) on cellular stress and iron metabolism in brown adipocytes. **(A)** Lipid peroxidation analysis with pre-lipid peroxidation (red, TRITC) and post-lipid peroxidation (green, FITC) from brown adipocytes after *Tfr1* knockdown (si*Tfr1*). Left: representative images of lipid peroxidation staining; right: lipid peroxidation fluorescence intensity presented as FITC/TRITC signal ratio. **(B)** ROS activity analysis with DCF (green) from brown adipocytes after *Tfr1* knockdown (si*Tfr1*). Left: representative images of ROS staining; right: ROS fluorescence intensity. **(C)** Gene expression analysis of iron metabolic and ferroptotic markers from brown adipocytes with or without *Tfr1* knockdown (si*Tfr1*). **(D)** Relative cellular viability analysis from brown adipocytes with or without *Tfr1* knockdown (si*Tfr1*). Data are presented as mean ± SEM. ^∗^*P* < 0.05; ^∗∗^*P* < 0.01. The results are representative of at least three independent experiments. NC, negative control.

Supplementary Figure 8 Single-cell sequencing data showing Tfr1-positive cell numbers from iWAT SVF of mice at different ages. ^∗^*P* < 0.05; ^∗∗^*P* < 0.01.

## Abbreviations

Tfr1: transferrin receptor
Ucp1: uncoupling protein 1
IR β: insulin receptor β
HFD: high-fat diet
DFO: deferoxamine
PFA: paraformaldehyde
BMI: body mass index
SVFs: stromal vascular fractions
iWAT: inguinal adipose tissue
eWAT: epididymal adipose tissue
BAT: brown adipose tissue
GWAS: genome-wide association studies
GO: gene ontology
MFs: molecular functions
CC: cellular component
KEGG: Kyoto Encyclopedia of Genes and Genomes.
