# Supplementary material for: Transferrin Receptor Functionally Marks Thermogenic Adipocytes
Source: Front Cell Dev Biol. 2020 Nov 5;8:572459. doi: 10.3389/fcell.2020.572459 (PMC7676909; doi:10.3389/fcell.2020.572459)
Supplement: Supplementary file 1 [file Table_1.DOC]

**Supplementary Table 1**

**Primer sequences for quantitative real-time PCR (qRT-PCR) .**

| **Gene name** | **Forward primer sequence 5'-3'** | **Reverse primer sequence 5'-3'** |
| --- | --- | --- |
| *m36b4* | GCTTCATTGTGGGAGCAGAC | ATGGTGTTCTTGCCCATCAG |
| *mTfr1* | GGCTACTTGGGCTATTGTAAAGG | CAGTTTCTCCGACAACTTTCTCT |
| *mUcp1* | GGCCCTTGTAAACAACAAAATAC | GGCAACAAGAGCTGACAGTAAAT |
| *mPgc1α* | ACCATGACTACTGTCAGTCACTC | GTCACAGGAGGCATCTTTGAAG |
| *mCidea* | TGACATTCATGGGATTGCAGAC | CGAGCTGGATGTATGAGGGG |
| *mElovl3* | TTCTCACGCGGGTTAAAAATGG | TCTCGAAGTCATAGGGTTGCAT |
| *mPrdm16* | CCACCAGCGAGGACTTCAC | GGAGGACTCTCGTAGCTCGAA |
| *mCpt1α* | TTGCCCTACAGCTCTGGCATTTCC | GCACCCAGATGATTGGGATACTGT |
| *mCox4β* | CTGCCCGGAGTCTGGTAATG | CAGTCAACGTAGGGGGTCATC |
| *mCytc* | AAATCTCCACGGTCTGTTCGG | GGGTATCCTCTCCCCAGGTG |
| *mMfn1* | AACCGAGAAGCTGCAGATGA | AGTTGGGCCACATCACACTC |
| *mMfn2* | AGGCCTTCCTCCTCACAGAG | GCAGGGTCAGTCAGGTCATCA |
| *mATPsyntβ* | GACATGGGCACAATGCAGG | GCAGGGTCAGTCAGGTCATCA |
| *mIrp1* | ACTCAAGATACGGACGCTTACC | GTTGCATGACATTCCAATTCAGG |
| *mIrp2* | CGGCACCAAGTATGATATTCTGC | AGGGCACTTCAACATTGCTCT |
| *mDmt1* | CAGCGAGACTTGGAGTGGTC | CACAGGATGATCCGTGGGA |
| *mMrckα* | AGAAGGATGCACGAGGCTATC | TTTCCAGGGCATATCTGTTGC |
| *mFht1* | CAAGTGCGCCAGAACTACCA | ACAGATAGACGTAGGAGGCATAC |
| *mFhl1* | CGTCAGAATTATTCCACCGAGG | GCCACGTCATCCCGATCAAA |
| *mPtgs2* | TTCCAATCCATGTCAAAACCGT | AGTCCGGGTACAGTCACACTT |
| *mChac1* | CTTGGTGGCTATGACACTAAGG | CCTCGGCAAGCAAGGATCTG |
| *mGpx4* | CATGCACGAATTCTCAGCCA | CATATCGGGCATGCAGATCG |
| *mFsp1* | CCTGGGGAAAAGGACAGATGAA | CATGGCAATGCAGGACAGGA |
| *mAcsl4* | CCTGAGGGGCTTGAAATTCAC | GTTGGTCTACTTGGAGGAACG |
| *mSrebf1* | TGACCCGGCTATTCCGTGA | CTGGGCTGAGCAATACAGTTC |
| *mSrebf2* | TGGGCGATGAGCTGACTCT | CAAATCAGGGAACTCTCCCAC |
| *mFabp4* | AAGGTGAAGAGCATCATAACCCT | TCACGCCTTTCATAACACATTCC |
| *mFasn* | GGAGGTGGTGATAGCCGGTAT | TGGGTAATCCATAGAGCCCAG |
| *mGck* | AGGAGGCCAGTGTAAAGATGT | CTCCCAGGTCTAAGGAGAGAAA |
| *mCox6a1* | CATGCTCAACGTGTTCCTCAA | AGAGGGTATGGTTACCGTCTC |
| *mSdha* | GCTTGCGAGCTGCATTTGG | TGTGATCGGGTAGGAAAGAGC |
| *mUqcrc1* | ACGCAAGTGCTACTTCGCA | CAGCGTCAATCCACACTCCC |
| *mNdufa6* | TCGGTGAAGCCCATTTTCAGT | CTCGGACTTTATCCCGTCCTT |
| *mAtp5b* | ACGTCCAGTTCGATGAGGGAT | TTTCTGGCCTCTAACCAAGCC |
| *mPpara* | AACATCGAGTGTCGAATATGTGG | CCGAATAGTTCGCCGAAAGAA |
| *mAcadl* | GCTTGGCATCAACATCGCAG | ATTCGCAATATAGGGCATGACAA |
| *mAcadm* | ATGCCTGTGATTCTTGCTGGA | ACATCTTCTGGCCGTTGATAAC |
| *m**Cpt1b* | TCTTCTTCCGACAAACCCTGA | GAGACGGACACAGATAGCCC |
| *mt-ND1* | GCGCTTTGAGACCTGGAAAAA | GGCCAGTGCGATAAAAGTTCAG |
| *mNdufa10* | ACCTTTCACTACCTGCGGATG | GTACCCAGGGGCATACTTGC |
| *mNdufs2* | CAGCCAGATATTGAATGGGCA | TGTTGGTCACCGCTTTTTCCT |
| *mNdufv1* | TTTCTCGGCGGGTTGGTTC | GGTTGGTAAAGATCCGGTCTTC |
| *mNdufv2* | GCAAGGAATTTGCATAAGACAGC | TAGCCATCCATTCTGCCTTTG |
| *mSdha* | GGAACACTCCAAAAACAGACCT | CCACCACTGGGTATTGAGTAGAA |
| *mSdhb* | AATTTGCCATTTACCGATGGGA | AGCATCCAACACCATAGGTCC |
| *mt-Cyb* | GGGCAGTCAGACAAGGATGTG | TCGTACACCTTATGATGCAGGA |
| *mCyc1* | CAGCTTCCATTGCGGACAC | GGCACTCACGGCAGAATGAA |
| *mUqpcrb* | GGCCGATCTGCTGTTTCAG | CATCTCGCATTAACCCCAGTT |
| *mCox5a* | ATGCCTGGGAATTGCGTAAAG | TGCGAACAGCACTAGCAAAAT |
| *mCox6a* | CTGCTCCCTTAACTGCTGGAT | GATTGTGGAAAAGCGTGTGGT |
| *mt-Atp6* | CTGGTGGCGGGTGCTTTAG | GCTACGTCTGGGATTCGATCT |
| *mAtp5a1* | TCTCCATGCCTCTAACACTCG | CCAGGTCAACAGACGTGTCAG |
| *mAtpif1* | GGTGTCTGGGGTATGAAGGTC | CCTTTTCTCGTTTTCCGAAGGC |
| *mPkm2* | *CGCCTGGACATTGACTCTG* | *GAAATTCAGCCGAGCCACATT* |
| *mHk2* | *ATGATCGCCTGCTTATTCACG* | *CGCCTAGAAATCTCCAGAAGGG* |
| *mPfk1* | *CATCGCCGTGTTGACCTCT* | *CCCGTGAAGATACCAACTCGG* |
| *mFasn* | *GGAGGTGGTGATAGCCGGTAT* | *TGGGTAATCCATAGAGCCCAG* |
