# Supplementary material for: Transferrin Receptor Functionally Marks Thermogenic Adipocytes
Source: Front Cell Dev Biol. 2020 Nov 5;8:572459. doi: 10.3389/fcell.2020.572459 (PMC7676909; doi:10.3389/fcell.2020.572459)
Supplement: Supplementary file 2 [file Table_2.DOC]

**Supplementary Table 2**

**Overlapped genes that featured high expressions in beige vs white adipocytes as well as iWAT under cold stimuli vs RT.**

| **Gene_name** | **Description** | **Fold Change (*C57*_iWAT/eWAT)** | **Fold Change (*129*_iWAT/eWAT)** | **Fold Change (iWAT_Cold/RT)** |
| --- | --- | --- | --- | --- |
| *Mb* | myoglobin | 255754.48 | 166944.91 | 30.45 |
| *Tpm1* | tropomyosin 1, alpha | 68493.15 | 324675.32 | 15.63 |
| *Tmem82* | transmembrane protein 82 | 4.71 | 2.90 | 13.36 |
| *2310042D19Rik* | RIKEN cDNA 2310042D19 gene | 3.38 | 3.25 | 11.60 |
| *Fabp3-ps1* | fatty acid binding protein 3, muscle and heart, pseudogene 1 | 19.30 | 6.33 | 9.52 |
| *Nr1d1* | nuclear receptor subfamily 1, group D, member 1 | 2.71 | 2.29 | 9.00 |
| *Ucp1* | uncoupling protein 1 (mitochondrial, proton carrier) | 157.31 | 3.63 | 6.99 |
| *Mreg* | melanoregulin | 30.07 | 17.01 | 6.31 |
| *Dnajc22* | DnaJ (Hsp40) homolog, subfamily C, member 22 | 11.96 | 4.91 | 5.92 |
| *Got1* | glutamate oxaloacetate transaminase 1, soluble | 2.84 | 2.52 | 4.42 |
| *Slc4a4* | solute carrier family 4 (anion exchanger), member 4 | 22675.74 | 3.76 | 4.15 |
| *Pfkm* | phosphofructokinase, muscle | 2.22 | 2.50 | 3.69 |
| *Tfr1* | transferrin receptor | 201207.24 | 5.62 | 3.61 |
| *S100b* | S100 protein, beta polypeptide, neural | 9.89 | 6.63 | 3.46 |
| *Mmd2* | monocyte to macrophage differentiation-associated 2 | 30.49 | 16.03 | 3.26 |
| *Ppargc1b* | peroxisome proliferative activated receptor, gamma, coactivator 1 beta | 27397.26 | 13642.56 | 3.25 |
| *Cidea* | cell death-inducing DNA fragmentation factor, alpha subunit-like effector A | 636.58 | 31.45 | 3.11 |
| *Ldhb* | lactate dehydrogenase B | 2.02 | 2.27 | 2.98 |
| *Gapdh* | glyceraldehyde-3-phosphate dehydrogenase | 5.01 | 3.22 | 2.76 |
| *Rmdn1* | regulator of microtubule dynamics 1 | 3.91 | 4.45 | 2.72 |
| *Acadvl* | acyl-Coenzyme A dehydrogenase, very long chain | 3.93 | 2.68 | 2.70 |
| *Suclg1* | succinate-CoA ligase, GDP-forming, alpha subunit | 2.97 | 58479.53 | 2.65 |
| *Tuba8* | tubulin, alpha 8 | 62.48 | 49.63 | 2.65 |
| *Aco2* | aconitase 2, mitochondrial | 4.04 | 5.62 | 2.52 |
| *Sucla2* | succinate-Coenzyme A ligase, ADP-forming, beta subunit | 2.90 | 2.00 | 2.49 |
| *Hadha* | hydroxyacyl-Coenzyme A dehydrogenase/3-ketoacyl-Coenzyme A thiolase/enoyl-Coenzyme A hydratase (trifunctional protein), alpha subunit | 3.63 | 318471.34 | 2.49 |
| *Rap1gap* | Rap1 GTPase-activating protein | 9.29 | 5.15 | 2.36 |
| *Cox17* | cytochrome c oxidase assembly protein 17 | 2.54 | 2.20 | 2.36 |
| *Impa2* | inositol (myo)-1(or 4)-monophosphatase 2 | 11.85 | 3.96 | 2.35 |
| *Cycs* | cytochrome c, somatic | 3.65 | 3.46 | 2.29 |
| *Hadhb* | hydroxyacyl-Coenzyme A dehydrogenase/3-ketoacyl-Coenzyme A thiolase/enoyl-Coenzyme A hydratase (trifunctional protein), beta subunit | 6.75 | 2.85 | 2.29 |
| *Uqcrc1* | ubiquinol-cytochrome c reductase core protein 1 | 3.37 | 2.36 | 2.24 |
| *Cox5a* | cytochrome c oxidase subunit Va | 3.43 | 2.57 | 2.18 |
| *Adck3* | aarF domain containing kinase 3 | 257731.96 | 5.62 | 2.18 |
| *Hspb6* | heat shock protein, alpha-crystallin-related, B6 | 4.67 | 5.10 | 2.15 |
| *Sdr39u1* | short chain dehydrogenase/reductase family 39U, member 1 | 3.69 | 3.25 | 2.12 |
| *Slc25a39* | solute carrier family 25, member 39 | 10.34 | 5.74 | 2.10 |
| *Uqcrc2* | ubiquinol cytochrome c reductase core protein 2 | 3.33 | 2.25 | 2.10 |
| *Mthfd2* | methylenetetrahydrofolate dehydrogenase (NAD+ dependent), methenyltetrahydrofolate cyclohydrolase | 2.54 | 2.10 | 2.07 |
| *Ndufab1* | NADH dehydrogenase (ubiquinone) 1, alpha/beta subcomplex, 1 | 3.25 | 3.87 | 2.07 |
| *Mrps36* | mitochondrial ribosomal protein S36 | 4.52 | 2.59 | 2.06 |
| *Dnaja3* | DnaJ (Hsp40) homolog, subfamily A, member 3 | 3.81 | 2.98 | 2.05 |
| *Ndufa5* | NADH dehydrogenase (ubiquinone) 1 alpha subcomplex, 5 | 3.21 | 3.27 | 2.03 |
| *Oplah* | 5-oxoprolinase (ATP-hydrolysing) | 3.65 | 48076.92 | 2.03 |
| *Ndufv2* | NADH dehydrogenase (ubiquinone) flavoprotein 2 | 3.53 | 2.48 | 2.02 |
| *Cox6b1* | cytochrome c oxidase, subunit VIb polypeptide 1 | 2.44 | 2.04 | 2.00 |

RT: Room Temperature, *C57*: *C57BL/6J*, *129*: *129/Sv*.
