# Supplementary figures and images for: Transferrin Receptor Functionally Marks Thermogenic Adipocytes
Source: Front Cell Dev Biol. 2020 Nov 5;8:572459. doi: 10.3389/fcell.2020.572459 (PMC7676909; doi:10.3389/fcell.2020.572459)

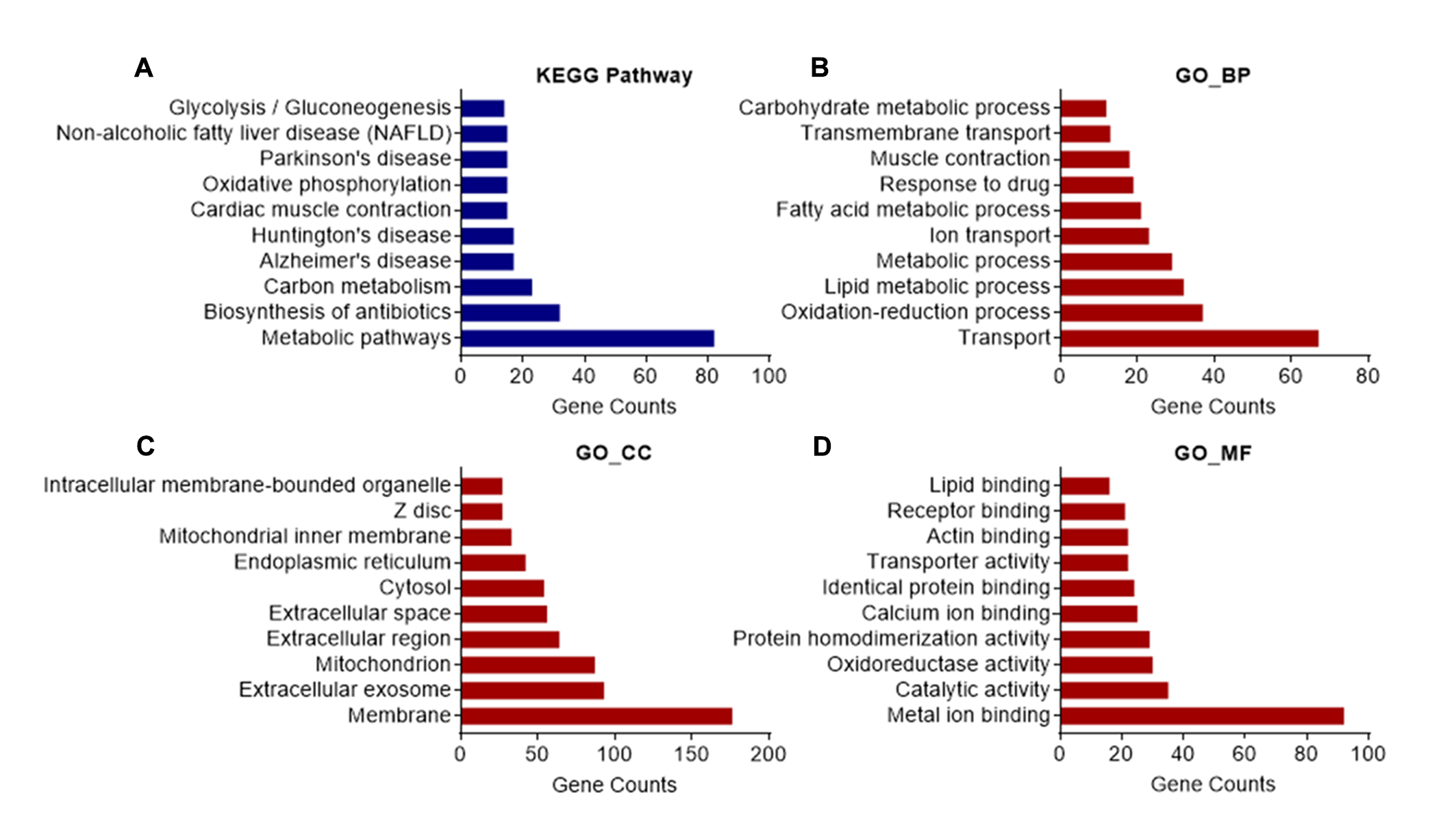

Supplement: Supplementary Figure 1 — In silico analysis of significantly altered genes in iWAT of mice with or without cold exposure. (A–D) KEGG pathway enrichment analysis (A) and GO analysis (B–D) of significantly altered genes in iWAT from mice under cold exposure or room temperature with top 10 different gene counts. [file Image_1.TIF]

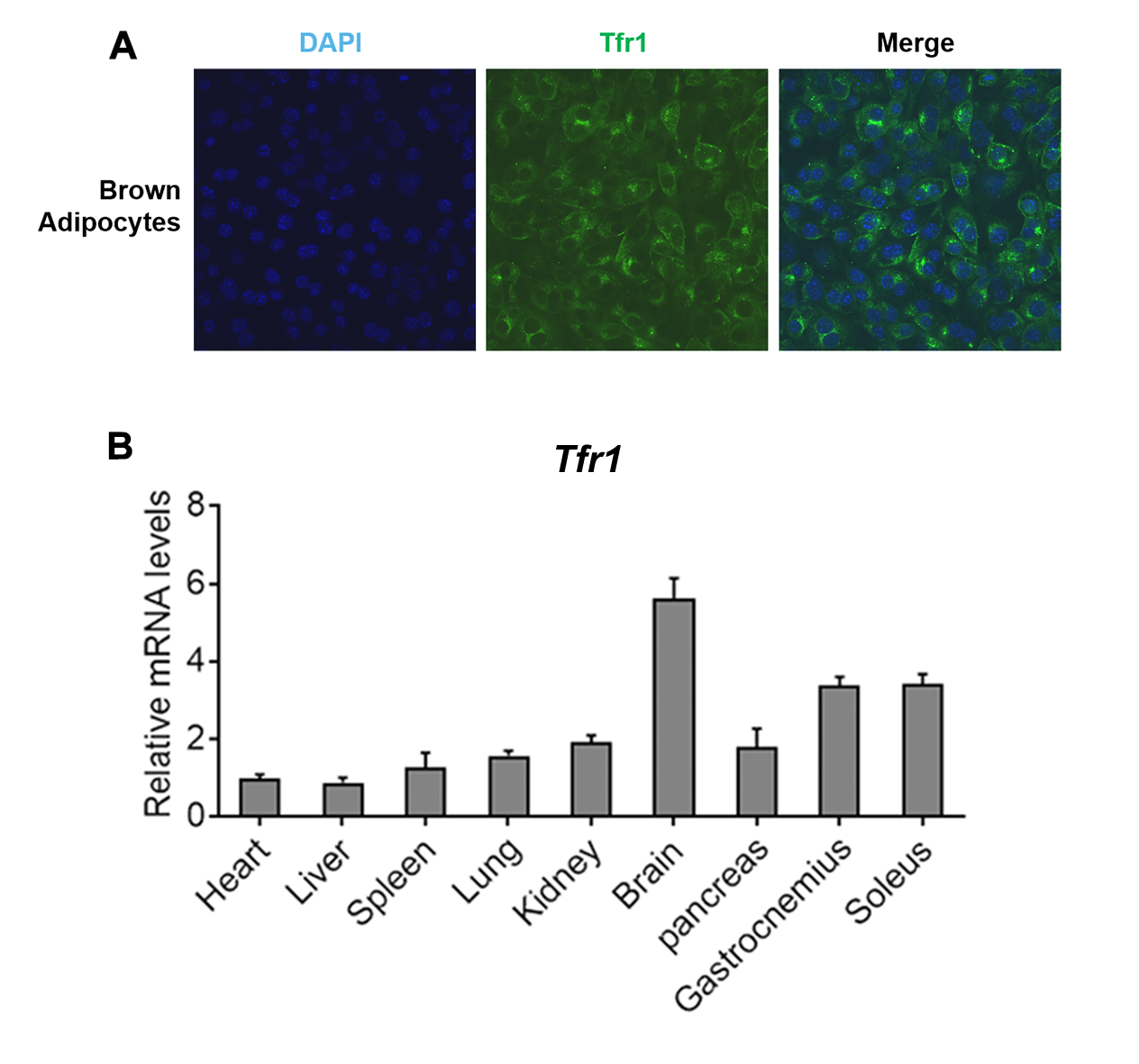

Supplement: Supplementary Figure 2 — Tfr1 expression pattern in multiple tissues. (A) Confocal images of immunofluorescence staining of Tfr1 (Phalloidin-Alexa488, green) and DAPI (blue). (B) Gene expression analysis of Tfr1 in heart, liver, spleen, lung, kidney, brain, pancreas, gastrocnemius, and soleus muscle of 2-month-old C57BL/6J mice. [file Image_2.TIF]

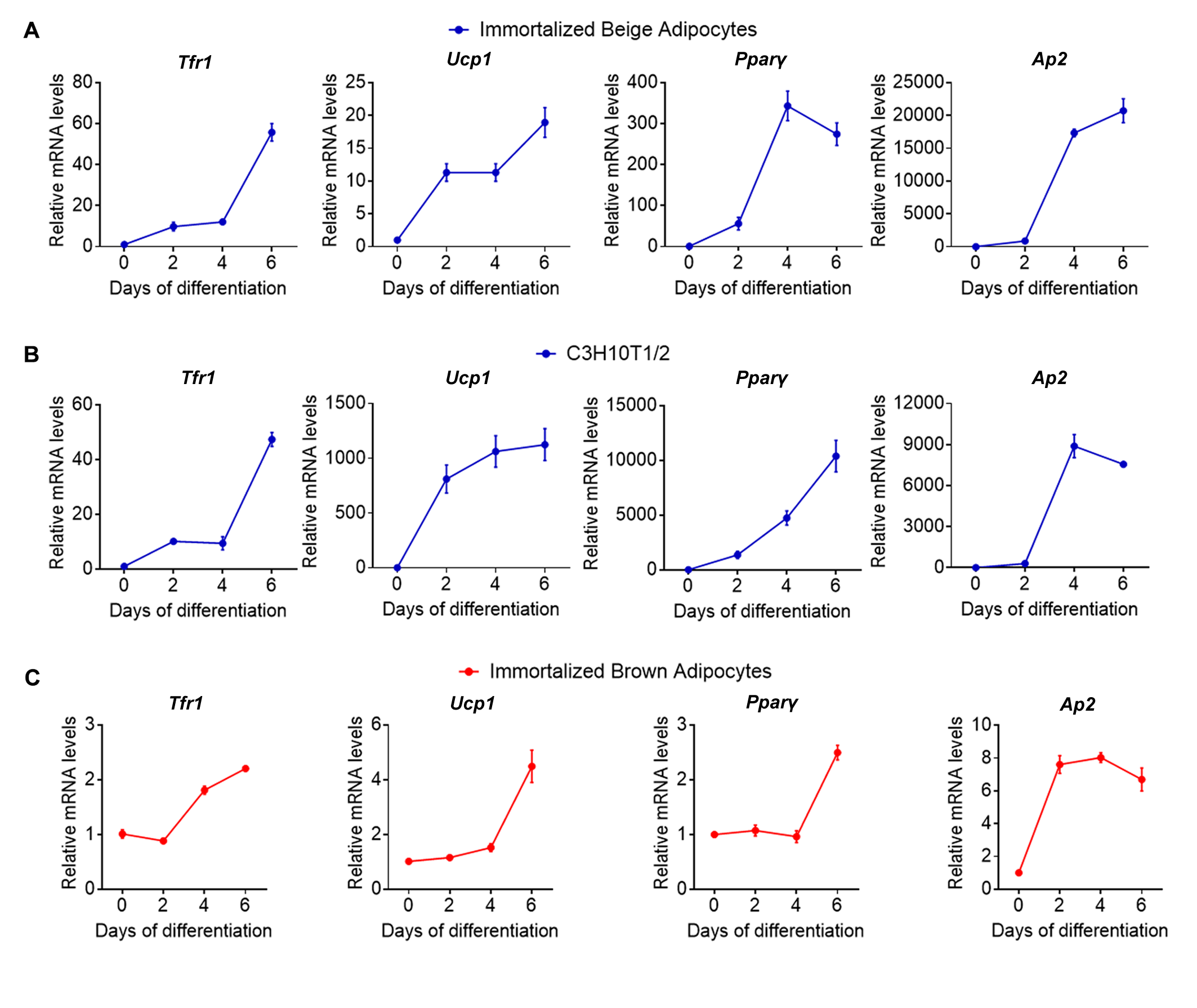

Supplement: Supplementary Figure 3 — Tfr1 expression levels during immortalized adipocyte differentiation. (A–C) Gene expression analysis of Tfr1, Ucp1, Pparγ, and Ap2 at indicated time point during differentiation in immortalized beige adipocytes (A), C3H10T1/2 adipocytes (B), and immortalized brown adipocytes (C). Data are presented as mean ± SEM. ∗P < 0.05; ∗∗P < 0.01. The results are representative of at least three independent experiments. [file Image_3.TIF]

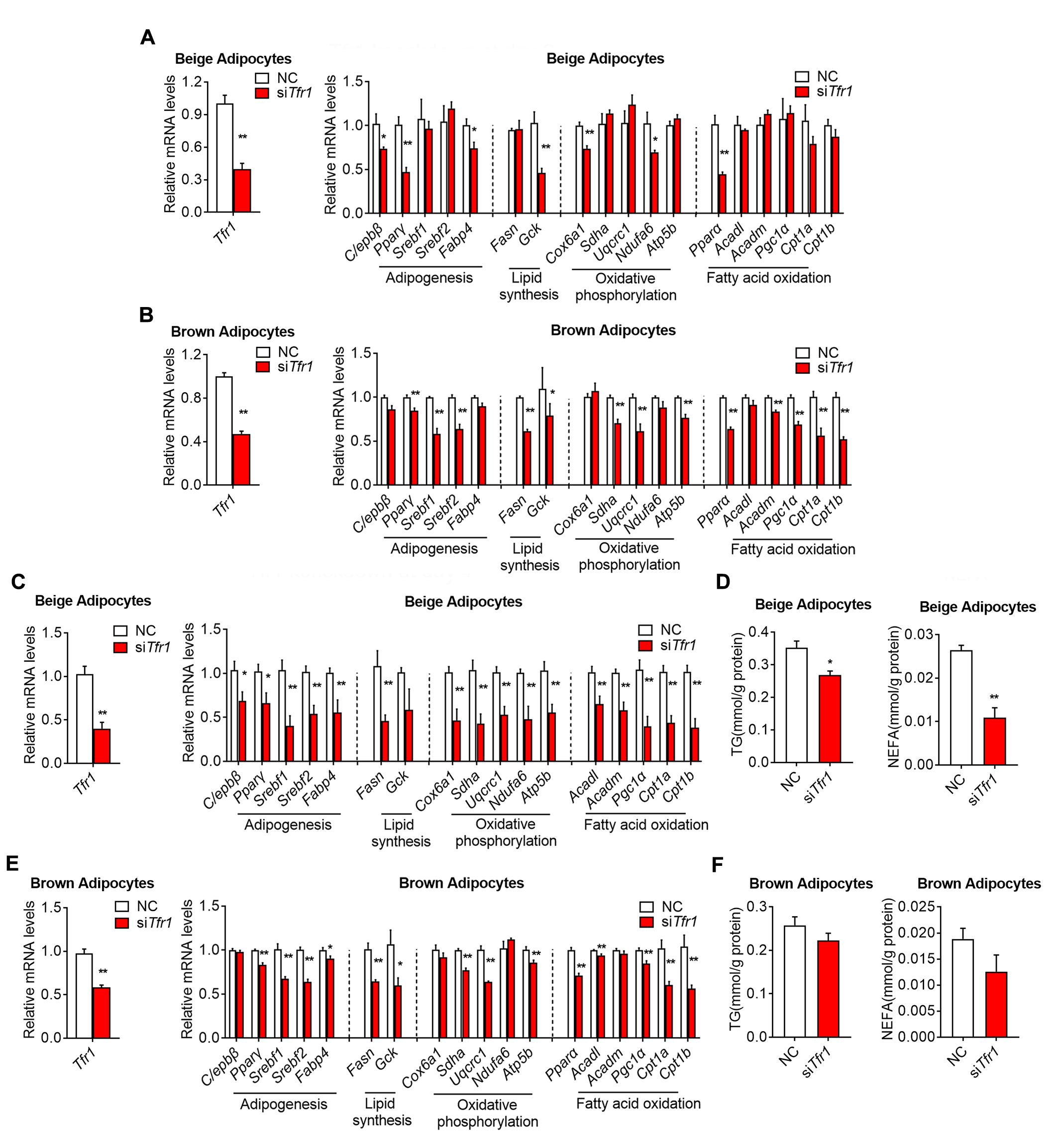

Supplement: Supplementary Figure 4 — The effect of Tfr1 knockdown (siTfr1) on lipid metabolism in thermogenic adipocytes. (A,B) Gene expression analysis of Tfr1, adipogenesis markers, lipid synthesis markers, oxidative phosphorylation markers and fatty acid oxidation markers from beige (A) and brown (B) adipocytes after 2 days of Tfr1 knockdown (siTfr1) on day 2 of differentiation. (C) Gene expression analysis of Tfr1, adipogenesis markers, lipid synthesis markers, oxidative phosphorylation markers, and fatty acid oxidation markers from beige adipocytes after 2 days of Tfr1 knockdown (siTfr1) on day 4 of differentiation. (D) TG and NEFA contents from beige adipocytes after 2 days of Tfr1 knockdown (siTfr1) on day 4 of differentiation. (E) Gene expression analysis of Tfr1, adipogenesis markers, lipid synthesis markers, oxidative phosphorylation markers, and fatty acid oxidation markers from brown adipocytes after 2 days of Tfr1 knockdown (siTfr1) on day 4 of differentiation. (F) TG and NEFA contents from brown adipocytes after 2 days of Tfr1 knockdown (siTfr1) on day 4 of differentiation. Data are presented as mean ± SEM. ∗P < 0.05; ∗∗P < 0.01. The results are representative of at least three independent experiments. NC, negative control. [file Image_4.TIF]

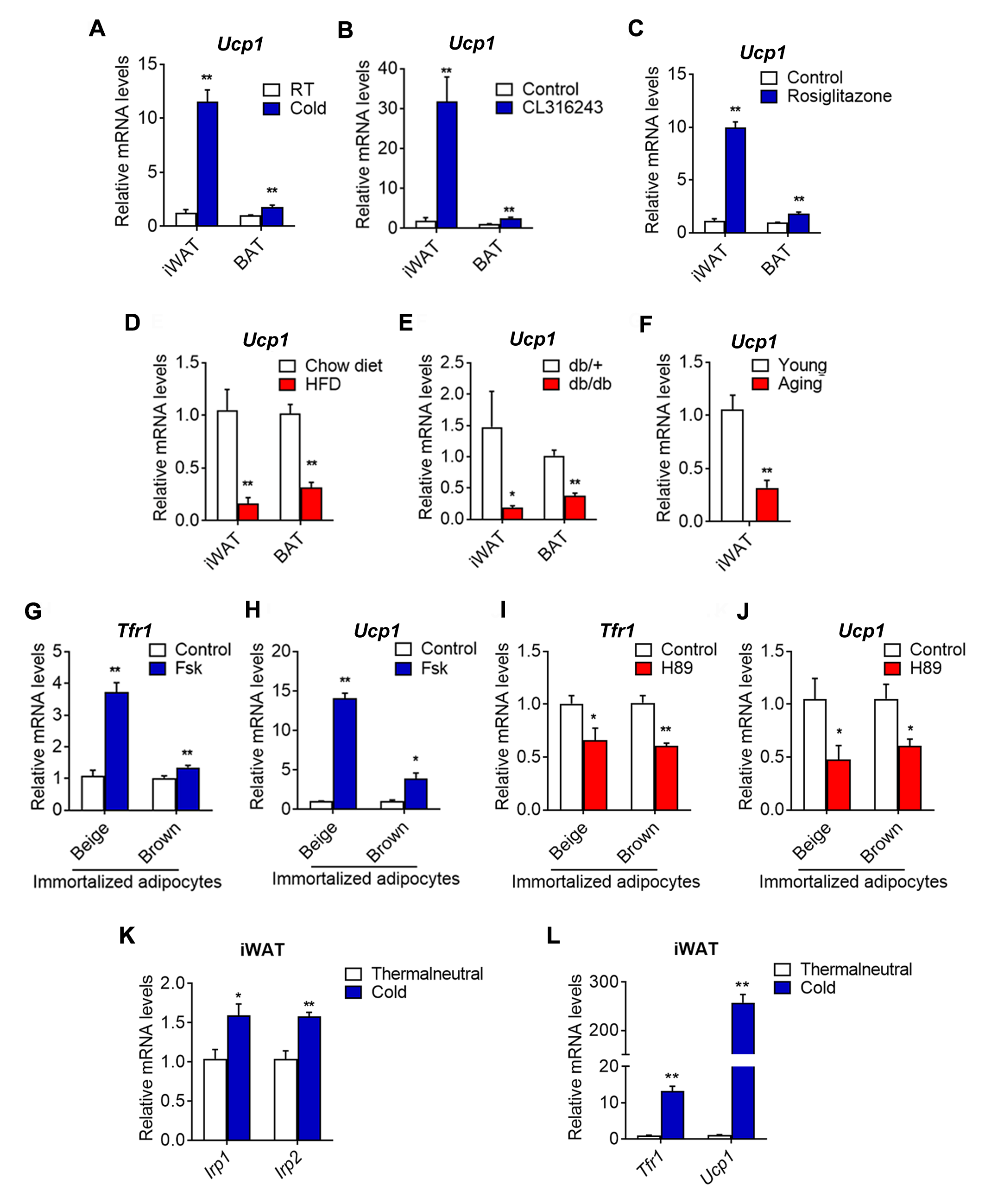

Supplement: Supplementary Figure 5 — Tfr1 expression level in iWAT and BAT under physiological and pathological stimuli. (A–C) Ucp1 mRNA levels in iWAT and BAT from mice under chronic cold condition (A), CL316243 injection (B) or Rosiglitazone treatment (C), compared to their individual control mice. (D–F) Ucp1 mRNA levels in iWAT and BAT from HFD diet fed (D), db/db (E) or aging (F) mice, compared to their individual control mice (Chow diet, db/+ or young mice). (G–J) Ucp1 (G,H) and Tfr1 (I,J) mRNA levels in immortalized adipocytes treated with or without Forskolin (FSK) and H89. (K–L) Irp1, Irp2 (K), Tfr1 and Ucp1 (L) mRNA levels in iWAT from mice under chronic cold condition, compared to thermalneutral condition. Data are presented as mean ± SEM. iWAT, inguinal adipose tissue; BAT, brown adipose tissue; RT, room temperature. ∗P < 0.05; ∗∗P < 0.01. The results are representative of at least three independent experiments. [file Image_5.TIF]

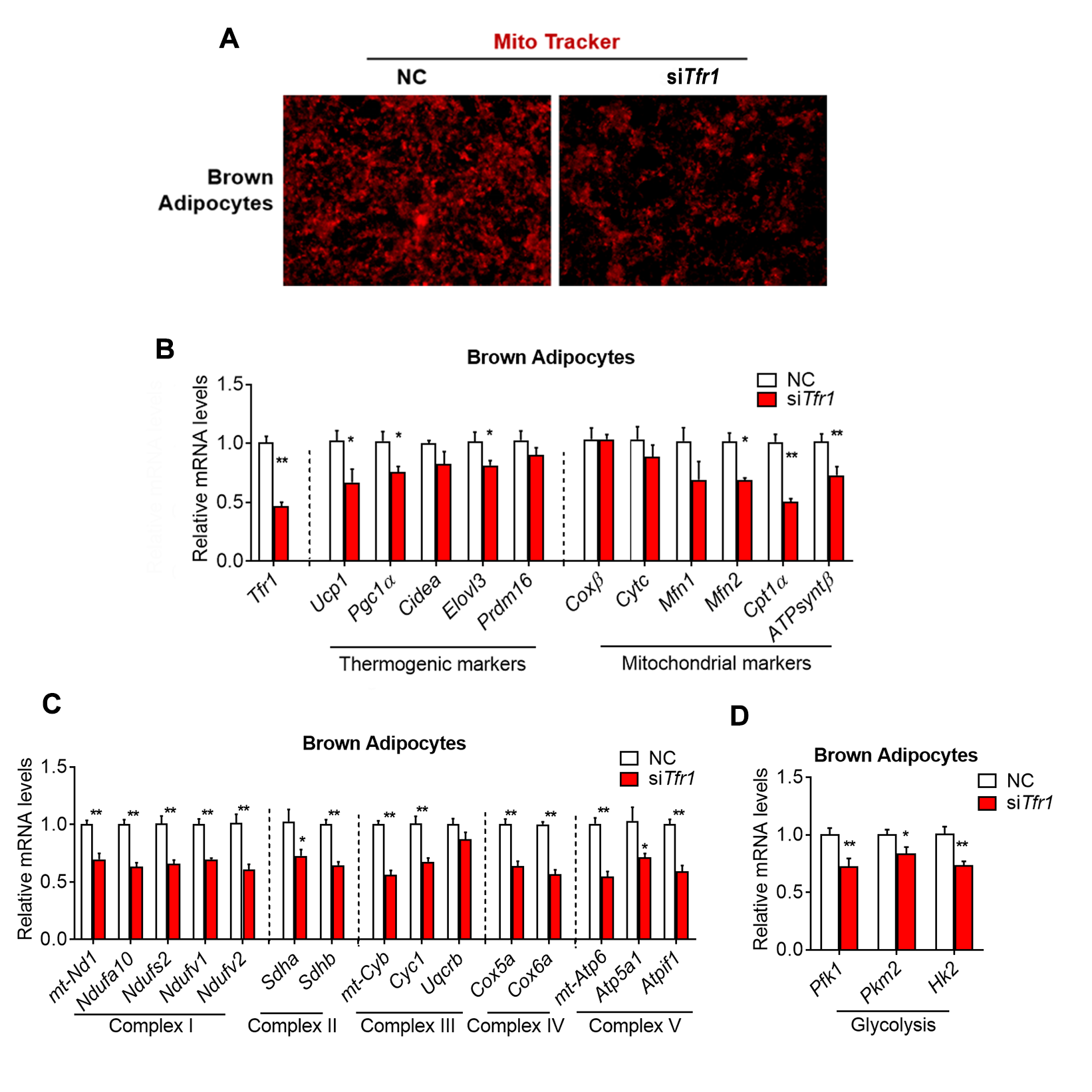

Supplement: Supplementary Figure 6 — The effect of Tfr1 knockdown (siTfr1) on mitochondrial function in brown adipocytes. (A) Mito Tracker staining of mature brown adipocytes with or without Tfr1 knockdown (siTfr1). (B) Gene expression analysis of Tfr1, thermogenic markers and mitochondrial markers from brown adipocytes with or without Tfr1 knockdown (siTfr1). (C) Gene expression analysis of five mitochondrial respiratory chain complex markers from brown adipocytes with or without Tfr1 knockdown (siTfr1). (D) Gene expression analysis of glycolysis markers from brown adipocytes with or without Tfr1 knockdown (siTfr1). Data are presented as mean ± SEM. ∗P < 0.05; ∗∗P < 0.01. The results are representative of at least three independent experiments. NC, negative control. [file Image_6.TIF]

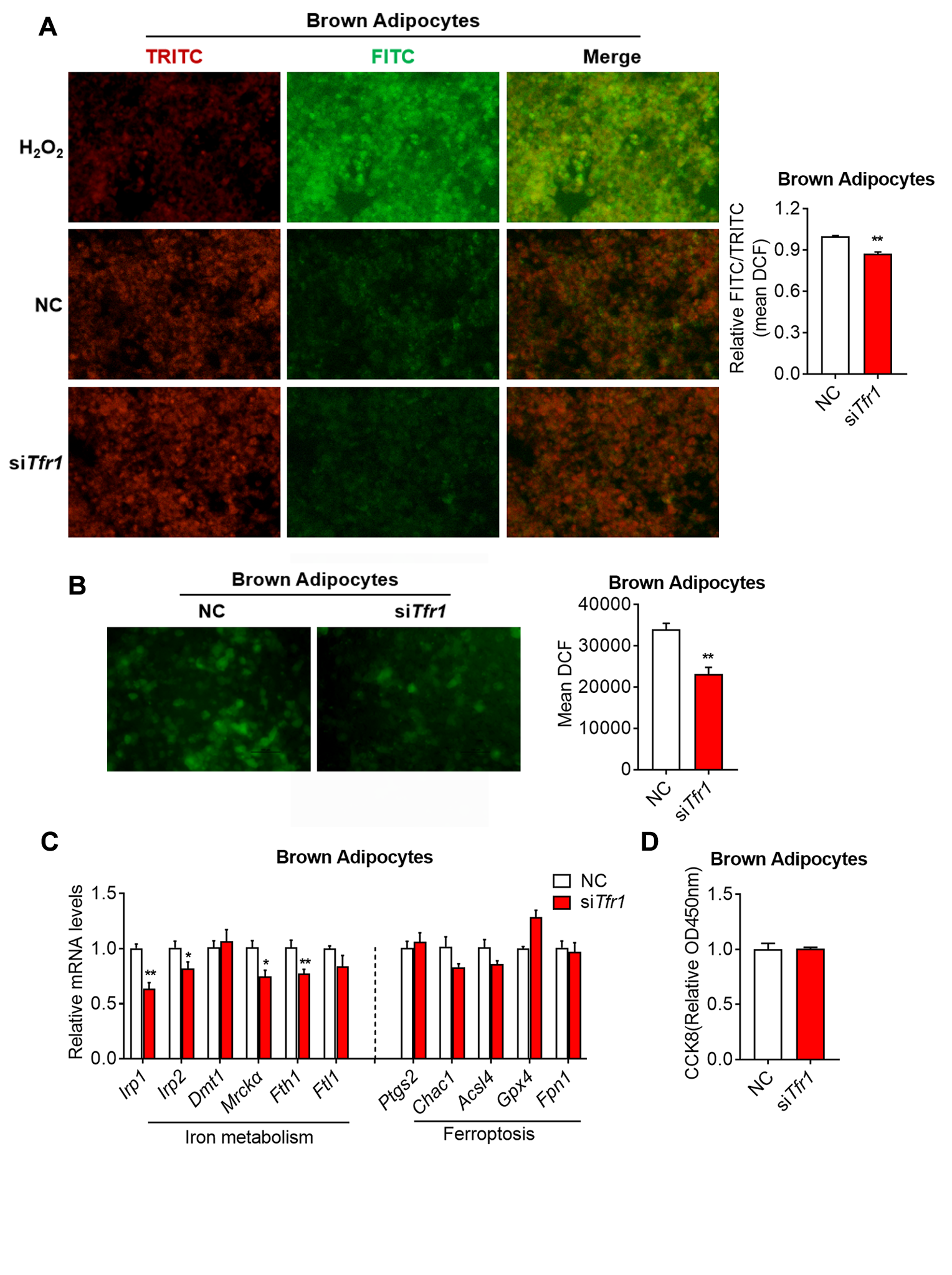

Supplement: Supplementary Figure 7 — The effect of Tfr1 knockdown (siTfr1) on cellular stress and iron metabolism in brown adipocytes. (A) Lipid peroxidation analysis with pre-lipid peroxidation (red, TRITC) and post-lipid peroxidation (green, FITC) from brown adipocytes after Tfr1 knockdown (siTfr1). Left: representative images of lipid peroxidation staining; right: lipid peroxidation fluorescence intensity presented as FITC/TRITC signal ratio. (B) ROS activity analysis with DCF (green) from brown adipocytes after Tfr1 knockdown (siTfr1). Left: representative images of ROS staining; right: ROS fluorescence intensity. (C) Gene expression analysis of iron metabolic and ferroptotic markers from brown adipocytes with or without Tfr1 knockdown (siTfr1). (D) Relative cellular viability analysis from brown adipocytes with or without Tfr1 knockdown (siTfr1). Data are presented as mean ± SEM. ∗P < 0.05; ∗∗P < 0.01. The results are representative of at least three independent experiments. NC, negative control. [file Image_7.TIF]

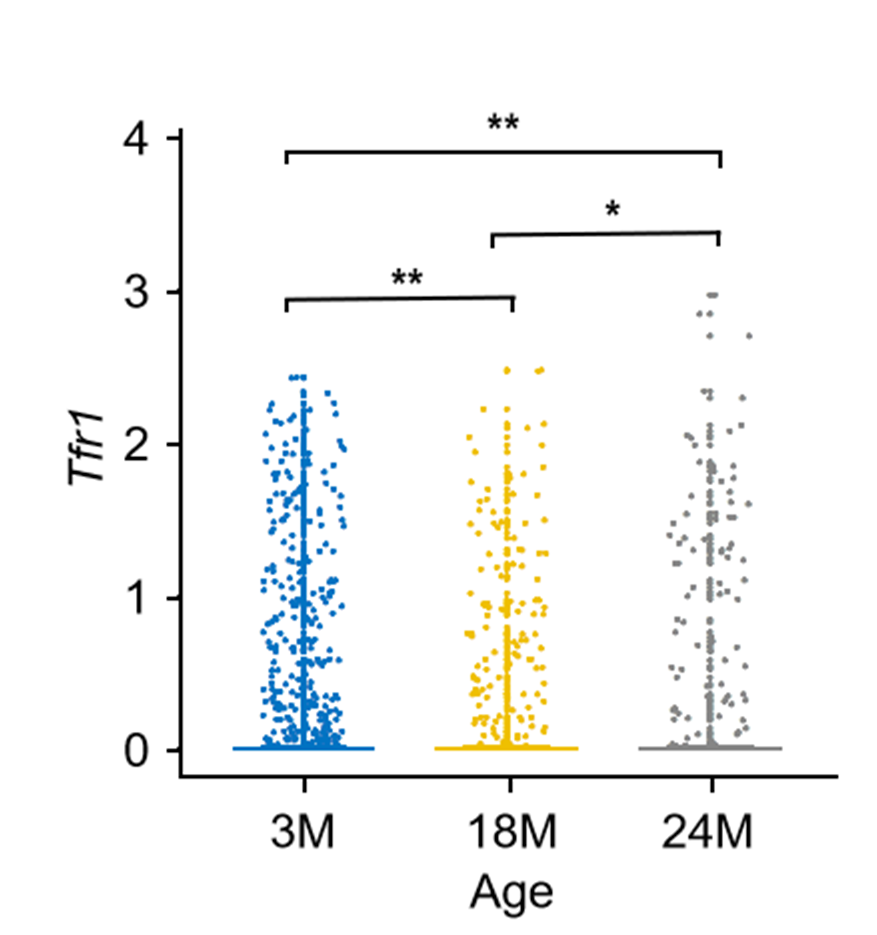

Supplement: Supplementary Figure 8 — Single-cell sequencing data showing Tfr1-positive cell numbers from iWAT SVF of mice at different ages. ∗P < 0.05; ∗∗P < 0.01. [file Image_8.TIF]
